# Rotenone Treatment Reveals a Role for Electron Transport Complex I in the Subcellular Localization of Key Transcriptional Regulators During T Helper Cell Differentiation

**DOI:** 10.3389/fimmu.2018.01284

**Published:** 2018-06-07

**Authors:** Emrah Ilker Ozay, Heather L. Sherman, Victoria Mello, Grace Trombley, Adam Lerman, Gregory N. Tew, Nagendra Yadava, Lisa M. Minter

**Affiliations:** ^1^Molecular and Cellular Biology Graduate Program, University of Massachusetts Amherst, Amherst, MA, United States; ^2^Department of Veterinary and Animal Sciences, University of Massachusetts Amherst, Amherst, MA, United States; ^3^Department of Biochemistry and Molecular Biology, University of Massachusetts Amherst, Amherst, MA, United States; ^4^Department of Microbiology, University of Massachusetts Amherst, Amherst, MA, United States; ^5^Department of Polymer Science and Engineering, University of Massachusetts Amherst, Amherst, MA, United States; ^6^Department of Biology, University of Massachusetts Amherst, Amherst, MA, United States; ^7^Pioneer Valley Life Sciences Institute, Springfield, MA, United States

**Keywords:** T cell metabolism, T cell differentiation, mitochondria, electron transport complex I, NADH:ubiquinone oxidoreductase, rotenone, Notch1

## Abstract

Recent advances in our understanding of tumor cell mitochondrial metabolism suggest it may be an attractive therapeutic target. Mitochondria are central hubs of metabolism that provide energy during the differentiation and maintenance of immune cell phenotypes. Mitochondrial membranes harbor several enzyme complexes that are involved in the process of oxidative phosphorylation, which takes place during energy production. Data suggest that, among these enzyme complexes, deficiencies in electron transport complex I may differentially affect immune responses and may contribute to the pathophysiology of several immunological conditions. Once activated by T cell receptor signaling, along with co-stimulation through CD28, CD4 T cells utilize mitochondrial energy to differentiate into distinct T helper (Th) subsets. T cell signaling activates Notch1, which is cleaved from the plasma membrane to generate its intracellular form (N1ICD). In the presence of specific cytokines, Notch1 regulates gene transcription related to cell fate to modulate CD4 Th type 1, Th2, Th17, and induced regulatory T cell (iTreg) differentiation. The process of differentiating into any of these subsets requires metabolic energy, provided by the mitochondria. We hypothesized that the requirement for mitochondrial metabolism varies between different Th subsets and may intersect with Notch1 signaling. We used the organic pesticide rotenone, a well-described complex I inhibitor, to assess how compromised mitochondrial integrity impacts CD4 T cell differentiation into Th1, Th2, Th17, and iTreg cells. We also investigated how Notch1 localization and downstream transcriptional capabilities regulation may be altered in each subset following rotenone treatment. Our data suggest that mitochondrial integrity impacts each of these Th subsets differently, through its influence on Notch1 subcellular localization. Our work further supports the notion that altered immune responses can result from complex I inhibition. Therefore, understanding how mitochondrial inhibitors affect immune responses may help to inform therapeutic approaches to cancer treatment.

## Introduction

CD4 T cells can differentiate into effector (Teff) or regulatory [induced regulatory T cell (iTreg)] cells, depending on extracellular cues that they experience at the time they are stimulated (1). During the differentiation process, activated T cells shift their metabolic status and, in this regard, certain metabolic processes have been shown to favor specific T cell programming (1–5). For instance, Teffs usually prefer to use aerobic glycolysis as an energy source, while iTregs are reported to be less glycolytic and primarily utilize fatty acid oxidation (FAO) and oxidative phosphorylation [OXPHOS; (2, 3, 6, 7)]. Studies show that Teffs and iTregs are unable to properly differentiate and function, unless they pass key metabolic checkpoints (8). Reports suggest iTregs fully oxidize the fatty acids and glucose in their mitochondria for ATP production, and using oligomycin to inhibit ATP synthase diminished T cell proliferation and function underscoring the importance of electron transport chain (ETC) activity in T cell activation (3, 9, 10). Given the fact that CD4 T cells display high mitochondrial content and activity, it is not surprising that mitochondrial metabolism may be a critical determinant in Th1, Th2, Th17, and iTreg differentiation (11). Moreover, mitochondrial metabolism has been shown to be essential to T cell plasticity, since inhibiting fatty acid synthesis induces a shift from Th17 toward iTreg differentiation, under Th17-polarizing conditions (1, 8, 10, 12). This inhibition led to decreased nuclear localization of RORγt and reduced binding to the *Il17a* enhancer locus, which ultimately resulted in the Th17-to-iTreg shift (12). Further reports showed the electron transport complex I (ETC-I) inhibitor, rotenone, selectively reduced Foxp3 expression and cytokine production during iTreg differentiation while minimally affecting T-bet and RORγt expression by Th1 and Th17 cells, respectively (13). Of note, rotenone had no effect on Foxp3 expression in fully differentiated iTregs, suggesting OXPHOS is plays a critical role during iTreg differentiation, but not maintenance, programs (13). ETC-I is the largest mitochondrial respiratory chain complex, contributing to ATP synthesis and mitochondrial membrane permeability (14). Rotenone treatment in T cells substantially affects multiple biological functions such as proliferation, cytokine production, and apoptosis (15–17). However, how ETC-I contributes, mechanistically, to T helper (Th) cell differentiation remains unclear.

Notch family proteins are type I transmembrane receptors involved in CD4 Th cell differentiation in response to extracellular polarizing cytokines (18, 19). The intracellular domain of Notch1 (N1ICD) has been shown to regulate T cell differentiation by signaling canonically or non-canonically, and by selectively binding to genes unique to each Th cell subset (18–20). It was shown that Notch1 can regulate the master transcription factors T-Bet, GATA3, RORγt, and Foxp3, as well as their target cytokine genes during Th cell differentiation (20–24). In addition, it has been reported that N1ICD translocates to the mitochondria and can regulate glycolysis, the TCA cycle, and OXPHOS (25, 26). In iTregs, mitochondrial localization of Notch1 was shown to be a critical determinant in fine-tuning differentiation and autophagy responses, thus, linking Notch1 signaling, mitochondrial metabolism, and T cell fate decisions (27).

Cancer cell mitochondrial metabolism may be an attractive therapeutic target, but the impact of mitochondrial inhibitors on immune cell activation and differentiation has not been elucidated. Here, we investigated the relationship between ETC-I activity and Notch1 signaling during Th cell differentiation and report that ETC-I activity influences Notch1 and transcription factor subcellular localization. We found that rotenone treatment increases mitochondrial association of Notch1 in Th2 and iTreg cell subsets and alters nuclear colocalization of Notch1 with Th-specific master transcription factors, especially with RORγt, by reducing Notch1 nuclear residence. Our data suggest that mitochondrial versus nuclear localization of Notch1 may be influenced by ETC-I activity to impact Th cell differentiation.

## Materials and Methods

### Materials

Rotenone ≥95% (Cas No.: 83-79-4) was purchased from Sigma Aldrich (St. Louis, MO, USA). Antibodies specific for mouse CD4 APC, CD4 FITC, Notch11 PE, GATA3 APC, and RORγt PE were purchased from eBioscience, Inc. (San Diego, CA, USA) and CD25 PECy7, T-bet APC, T-bet PECy7, and Foxp3 AF488 were purchased from BioLegend (San Diego, CA, USA). Notch1 FITC was purchased from GeneTex, Inc. (Irvine, CA, USA). Unconjugated pyruvate dehydrogenase kinase 1 (PDHK1) (written as PDK1) (Clone: 4A11F5), PDH-E1α (Clone: D-6), and Tubulin AF647 (Clone: A-6) were purchased from Santa Cruz Biotechnology, Inc. (Santa Cruz, CA, USA). Phospho PDH-E1α (Ser232) was purchased from MilliporeSigma (Burlington, MA, USA). For secondary antibodies, BV510TM donkey anti-rabbit IgG (minimal x-reactivity) (Clone: Poly4064) and BV421TM goat anti-mouse IgG (minimal x-reactivity) (Clone: Poly4053) were purchased from BioLegend. Recombinant IL-2, IFNγ, IL-4, IL-17A, and IL-10 (carrier-free) were purchased from BioLegend. Coating and detection antibodies for IL-2, IFNγ, IL-4, and IL-17A were purchased from BD Biosciences (Billerica, MA, USA) and for IL-10, from BioLegend. For live/dead staining Zombie Violet Fixable Viability Kit was purchased from BioLegend and 7-aminoactinomycin D (7-AAD) staining solution was purchased from BD Biosciences For imaging purposes, MitoTracker Red CMXRos and DRAQ5 were purchased from Thermo Fisher Scientific (Waltham, MA, USA). Purified anti-CD3ε (145-2C11) and anti-CD28 (37.51) were purchased from BioLegend. Flow cytometric data were acquired using an LSR II Flow Cytometer, LSRFortessa™ 5 laser (Becton Dickinson, Canaan, CT, USA) and analyzed using DIVA 7.0 software (Becton Dickinson) or FlowJo v10.0 (Tree Star, Ashland, OR, USA). Imaging flow cytometry data were acquired using AMNIS ImageStream X Mark II Imaging Flow Cytometer and analyzed using IDEAS software (EMD Millipore, Billerica, MA, USA).

### Animals

All mouse protocols were approved by the Institutional Animal Care and Use Committee of the University of Massachusetts Amherst. C57BL/6J mice were obtained from The Jackson Laboratory (Bar Harbor, ME, USA). Offspring between the ages of 9 and 12 weeks old were used in experiments.

### CD4 T Cell Isolation and Rotenone Treatment

Mouse spleens were harvested from C57BL/6J mice and passed through a cell strainer. Red blood cells were lysed in ACK Lysis Buffer, pH 7.2 (150 mM NH_4_Cl, 10 mM KHCO_3_, 0.1 mM EDTA) to obtain bulk splenocytes. Subsequently, primary CD4 T cells were isolated using BD IMag CD4 magnetic particles (Clone GK1.5) (BD Biosciences). CD4 T cells were treated with 20 µM rotenone for 2 h at 37°C prior to stimulating them on anti-CD3ε plus anti-CD28-coated wells.

### Cellular Viability Assay

Rotenone-treated primary mouse splenic CD4 T cells were harvested at designated time points and stained with 7-AAD (eBioscience) for 15 min at room temperature followed by centrifugation for 5 min. Cells were resuspended in 0.2% BSA in PBS and analyzed by flow cytometry.

### Flow Cytometric Analysis of T Cell Activation

Primary mouse splenic CD4 T cells were pretreated with 20 µM rotenone for 2 h at 37°C prior to stimulating in anti-CD3ε + anti-CD28-coated wells in 5% CO_2_ at 37°C. After 24 h of stimulation, cells were harvested and surface-stained with CD4 APC and CD25 PECy7. Cells were then fixed and permeabilized by using the Foxp3/Transcription Factor Staining Buffer Set (Thermo Fisher Scientific). They were intracellularly stained for Notch1 PE (Clone: mN1A) and analyzed *via* flow cytometry.

### Enzyme-Linked Immunosorbent Assay (ELISA)

Primary mouse splenic CD4 T cell culture supernatants were collected at designated time points and analyzed for cytokine secretion. 96-well Maxisorp plates were coated overnight at 4°C with the appropriate capture antibody (anti-mouse IFNγ, anti-mouse IL-2, anti-IL-4, anti-IL-17A, and anti-IL-10; BD Biosciences). Non-specific protein binding was prevented by blocking wells with 5% BSA in PBS for 3 h at room temperature. Culture supernatants and standards (recombinant IFNγ and IL-2, carrier-free) were diluted appropriately and added to wells. The plate was incubated overnight at 4°C, with continuous rocking. Biotinylated detection antibodies were added to wells followed by TMB substrate reagents (BD Biosciences) at a 1:1 ratio. Color development was monitored, and the reaction was terminated by the addition of stop solution (2N H_2_SO_4_). Absorbance was read at 450 nm using a microplate reader. Cytokine concentrations were determined relative to the standard curves generated.

### *In Vitro* Mouse T Cell Differentiation Into Th1, Th2, and Th17 Subsets

Primary mouse splenic CD4 T cells were pretreated with 20 µM rotenone for 2 h at 37°C and then they were resuspended in complete RPMI-1640 media (10% fetal bovine serum, 100 U/mL penicillin–streptomycin, 1 mM sodium pyruvate, 2 mM L-glutamine, β-mercaptoethanol). For polarizations into Th1, Th2, and Th17 subsets, specific cytokine cocktails were added into each cell suspension [Th1: 10 µg/mL anti-IL-4 (BioLegend) + 1 ng/mL recombinant IL-12 (eBioscience, Inc.), Th2: 10 µg/mL anti-IFNγ (BD Biosciences) + recombinant IL-4 (eBioscience, Inc.), Th17: 10 µg/mL anti-IL-4 (BioLegend) + 10 µg/mL anti-IFNγ (BD Biosciences) + 20 ng/mL recombinant IL-6 (BioLegend) + 5 ng/mL TGF-β (BioLegend)]. The cells were incubated for 24, 48, 72, and 96 h for further analysis.

### *In Vitro* Mouse iTreg Differentiation

Primary mouse bulk splenocytes were incubated with CD4 T cell enrichment antibody cocktail (BD Biosciences) for 15 min on ice, then incubated with Streptavidin Particles Plus—DM (BD Biosciences) for 30 min on a rotator at 4°C. The negative fractions from magnetic separation were collected in a conical tube. Later, biotin-conjugated anti-CD25 (2.5 μg) was added into the negative fractions and incubated on ice for 30 min. Subsequently, they were incubated with Streptavidin Particles Plus—DM (BD Biosciences) for 30 min on a rotator at 4°C and separated magnetically to obtain CD4^+^ CD25^−^ T cells in the negative fraction. The cells were resuspended in complete RPMI-1640. IL-2 (135 U/mL) and TGF-β (20 ng/mL) were added into the cell suspension to polarize them toward iTregs. The cells were incubated for 24, 48, 72, and 96 h for further analysis.

### Imaging Flow Cytometry

Each Th subset was harvested at the indicated time points following polarization. Mitochondria were visualized using 300 nM MitoTracker Red CMXRos and nuclei using DRAQ5 (1:1,000). Notch1 FITC was used to stain the cells intracellularly. Later, 1,000 cells were imaged, and fluorescent intensities were quantified using an AMNIS ImageStream X Mark II Imaging Flow Cytometer at 60× magnification. To determine nuclear localization of desired proteins, the nuclear localization wizard was applied in the IDEAS software upon masking the nuclear area (Intensity mask: Ch05-DRAQ5 staining). For mitochondrial localization of Notch1, the colocalization wizard was used to determine the bright detail similarity between MitoTracker Red signal and Notch1 FITC signal. A similar protocol was followed to assess localization of Th-specific master transcription factors in the mitochondria and nuclei of imaged cells. To visualize Th17-polarized cells treated with rotenone or dichloroacetate (DCA), primary mouse splenic CD4 T cells were pretreated with 20 µM rotenone for 2 h or 1 mM DCA (left in the cell suspension throughout the experiment) at 37°C and then they were resuspended in complete RPMI-1640 media (10% fetal bovine serum, 100 U/mL penicillin–streptomycin, 1 mM sodium pyruvate, 2 mM l-glutamine, β-mercaptoethanol). Later, cells were polarized into Th17 subset by adding 10 µg/mL of anti-IL-4 (BioLegend) + 10 µg/mL of anti-IFNγ (BD Biosciences) + 20 ng/mL of recombinant IL-6 (BioLegend) + 5 ng/mL of TGF-β (BioLegend) and cultured for 72 h on anti-CD3ε + anti-CD28-coated wells. At 72 h, cells were harvested and stained for mitochondria with MitoTracker Red CMXRos, cytosol with Tubulin AF647, and nuclei with propidium iodide for imaging flow cytometry *via* AMNIS ImageStream X Mark II Imaging Flow Cytometer at 60× magnification. Th17-polarized cells were gated as RORγt-positive cells and analyzed for localizations for PDHK1 (followed by secondary BV510 anti-rabbit IgG), total PDH-E1α (followed by secondary BV510 anti-rabbit IgG), pPDH-E1α (Ser232) (followed by secondary BV421 anti-mouse IgG), RORγt PE, and Notch1 FITC. The data were analyzed *via* IDEAS software.

### Statistical Analysis

Data are the mean ± SEM; all experiments were repeated at least two or three times. Unpaired, two-tailed Student’s *t*-test and two-way ANOVA with post-Bonferroni test were applied for statistical comparison by using GraphPad Prism 5 software. *p* Values of ≤0.05 were considered significant.

## Results

### Rotenone Treatment Reduces T Cell Activation Upon Anti-CD3 Plus Anti-CD28 Stimulation

**Figure 1:**
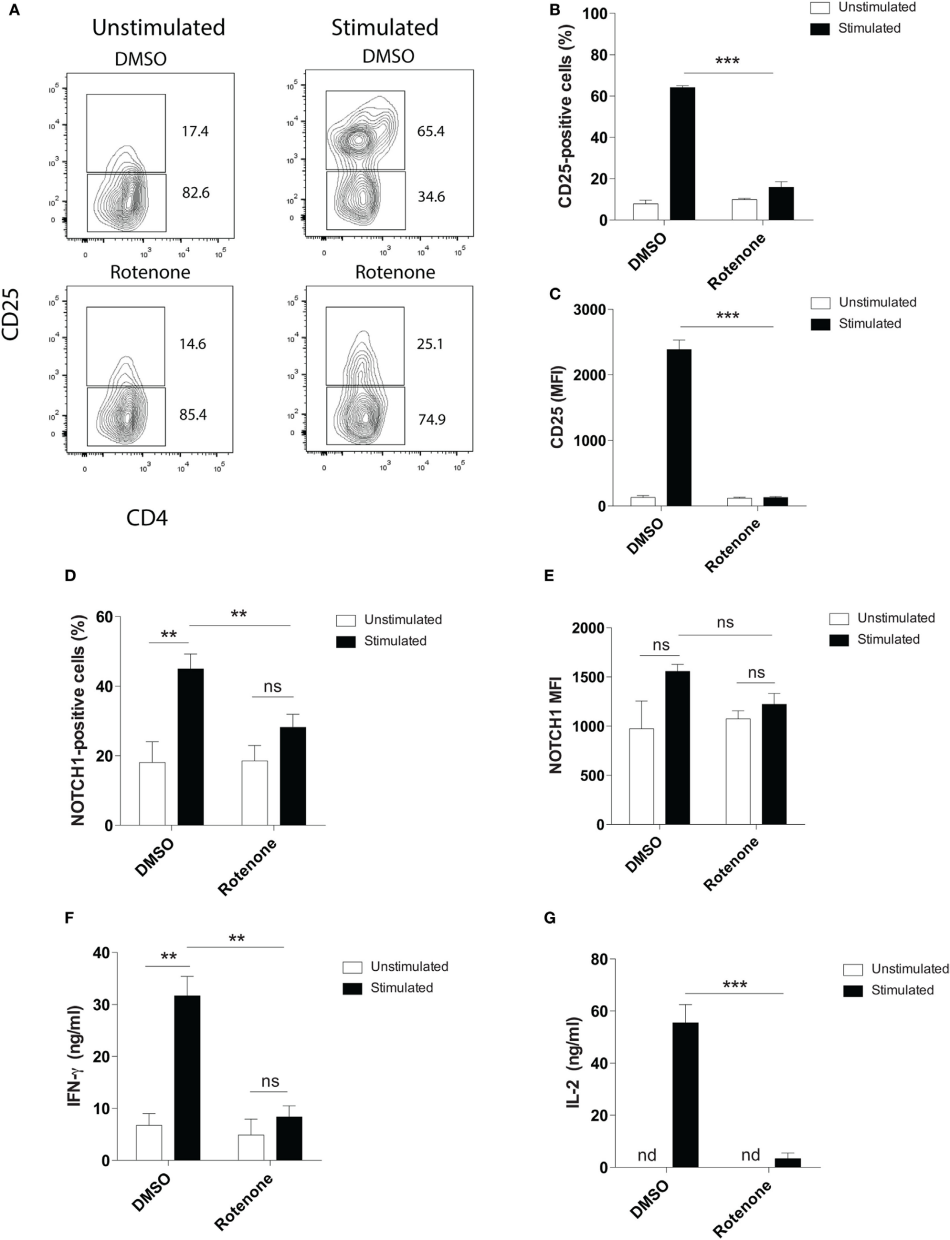
Rotenone treatment reduces T cell activation upon anti-CD3ε plus anti-CD28 stimulation. Mouse splenic CD4 T cells were left untreated or treated with 20 µM rotenone for 2 h, then stimulated with plate-bound anti-CD3ε plus anti-CD28 for 24 h. CD25 and Notch1 levels were measured as read-outs of T cell activation *via* flow cytometry. **(A)** Representative contour plot showing CD25-expressing cells left unstimulated or stimulated in the presence or absence of rotenone for 24 h. The **(B)** percent CD25-positive, and **(C)** median fluorescence intensity (MFI) of CD25, as well as the **(D)** percent Notch1-positive **(E)** MFI of Notch1 on CD4 T cells, cultured as described above. At the end of 24 h of stimulation, culture supernatants were collected, and standard enzyme-linked immunosorbent assay techniques were used to quantify secreted **(F)** IFNγ and **(G)** IL-2. Data represent the mean ± SEM of three independent experiments. *p < 0.05; **p < 0.01; ***p < 0.001; calculated using an unpaired, two-tailed Student’s *t*-test.

Mitochondrial metabolism has been previously implicated in antigen-specific T cell activation *in vivo*, through the production of reactive oxygen species by ETC-III (9). To investigate whether ETC-I also functions to mediate CD4 T cell activation, we pretreated primary mouse splenic CD4 T cells with the ETC-I inhibitor, rotenone (20 µM), for 2 h at 37°C, then stimulated cells on anti-CD3ε- plus anti-CD28-coated plates for 24 h. Upon stimulation, the high affinity IL-2 receptor, CD25, is upregulated on CD4 T cells through a Notch1-dependent mechanism (28, 29). Using conventional flow cytometry, which quantifies total protein, but not its subcellular localization, we evaluated CD25 expression following T cell stimulation and found that, compared with cells incubated only with vehicle control, DMSO, significantly fewer cells upregulated CD25 following rotenone treatment, and the cells that did express CD25 showed markedly reduced levels of protein expression (Figures 1A–C). We and others have shown that stimulating CD4 T cells *in vitro* generates the cleaved, signaling competent, intracellular domain of Notch1, N1ICD (20, 30). Therefore, we measured the percentage of cells that expressed N1ICD, as well as the total N1ICD they expressed on a per cell basis. Consistent with CD25 results, we noted reduced percentages of CD4 T cells that expressed N1ICD, following rotenone treatment (Figure 1D), but interestingly, the overall expression of N1ICD did not appear to be significantly different between rotenone- and DMSO-treated cells (Figure 1E). However, when we analyzed the culture supernatants from cells left untreated or treated with rotenone, we noted that secretion of IFNγ and IL-2, in the culture supernatants was reduced (Figures 1F,G). Thus, rotenone treatment reduces CD4 T cell activation potential upon anti-CD3ε plus anti-CD28 stimulation as measured by CD25 expression and secretion of IFNγ and IL-2, although the cellular levels of N1ICD did not appear to differ between cells treated with rotenone or DMSO.

### Rotenone Treatment Alters the Kinetics of Th17 and iTreg Cell Differentiation

**Figure 2:**
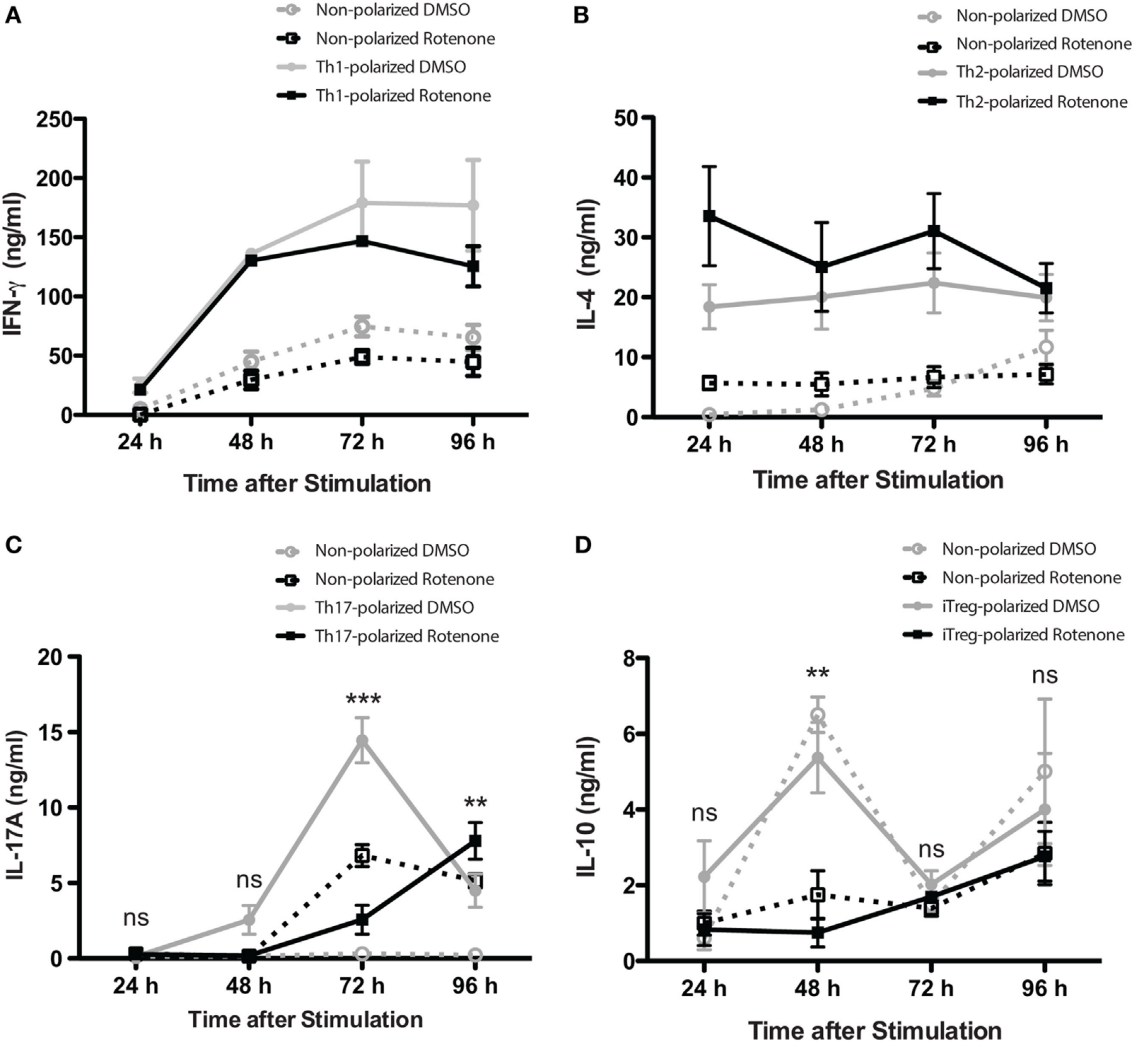
Rotenone treatment alters the kinetics of Th17 and induced regulatory T cell (iTreg) cell differentiation. Mouse splenic CD4 T cells and CD4^+^CD25^−^ T cells were used for T helper (Th) cell and iTreg differentiation, respectively. Cells were left untreated or treated with 20 µM rotenone for 2 h and then stimulated with plate-bound anti-CD3ε plus anti-CD28 for 24, 48, 72, and 96 h in the presence of specific Th cell polarization conditions. At the indicated time points, cell supernatants were collected, and signature cytokines were quantified using standard enzyme-linked immunosorbent assay techniques for **(A)** IFNγ by Th1-polarized cells, **(B)** IL-4 by Th2-polarized cells, **(C)** IL-17A by Th17-polarized cells, and **(D)** IL-10 by iTreg-polarized cells. Data represent the mean ± SEM of three independent experiments. *p < 0.05; **p < 0.01; ***p < 0.001; calculated using two-way ANOVA with post-Bonferroni test applied.

Studies have highlighted how metabolic pathways are selectively utilized during Th cell differentiation (31, 32). Teff cells rely on glucose as their main energy source, while iTregs will oxidize fatty acids to produce the energy they need. Rotenone acts to block NADH → NAD, a process important both in glycolytic and OXPHOS pathways. Therefore, we sought to further investigate whether rotenone treatment would alter the differentiation potential of Th cells. We performed polarization assays to differentiate Th1, Th2, Th17, and iTreg cells *in vitro*. CD4 T cells were isolated from mouse spleens and left untreated or treated with rotenone (20 µM) for 2 h at 37°C. Cells were stimulated with anti-CD3ε and anti-CD28 in the presence of specific combinations of antibodies and cytokines to promote their differentiation toward distinct Th cell lineages. We harvested polarized cells 24, 48, 72, and 96 h after plating and used standard ELISA techniques to analyze the secretion kinetics of their signature cytokines: Th1:IFNγ, Th2:IL-4, Th17:IL-17A, and iTreg:IL-10. Overall, rotenone treatment did not affect cellular viability over their time course (Figures S1A–D in Supplementary Material). Interestingly, we observed that rotenone affected the cytokine secretion of distinct Th subsets, but with different kinetics. We did not detect significant difference in IFNγ secretion by Th1-polarized cells or in IL-4 secretion by Th2-polarized cells over time, regardless of whether the cells were polarized in the absence or presence of rotenone (Figures 2A,B). However, we found IL-17A levels were greatly diminished by 72 h in Th17-polarized cells exposed to rotenone treatment (Figure 2C). IL-10 secretion by iTregs was also significantly lower in rotenone-treated cells, compared with those cultured in DMSO. However, we noted that the kinetics of reduced IL-10 production were different from Th17-polarized cells, with the maximum differences in effects on IL-10 production occurring 48 h after stimulation (Figure 2D). Altogether, these findings suggest that rotenone treatment uniquely modifies the kinetics of cytokine production in Th cells during *in vitro* polarization, with Th17 and iTreg cells being particularly responsive to ETC-1 inhibition.

### Notch1 Expression and Cellular Localization in Th Cells Is Differentially Affected by Rotenone Treatment

**Figure 3:**
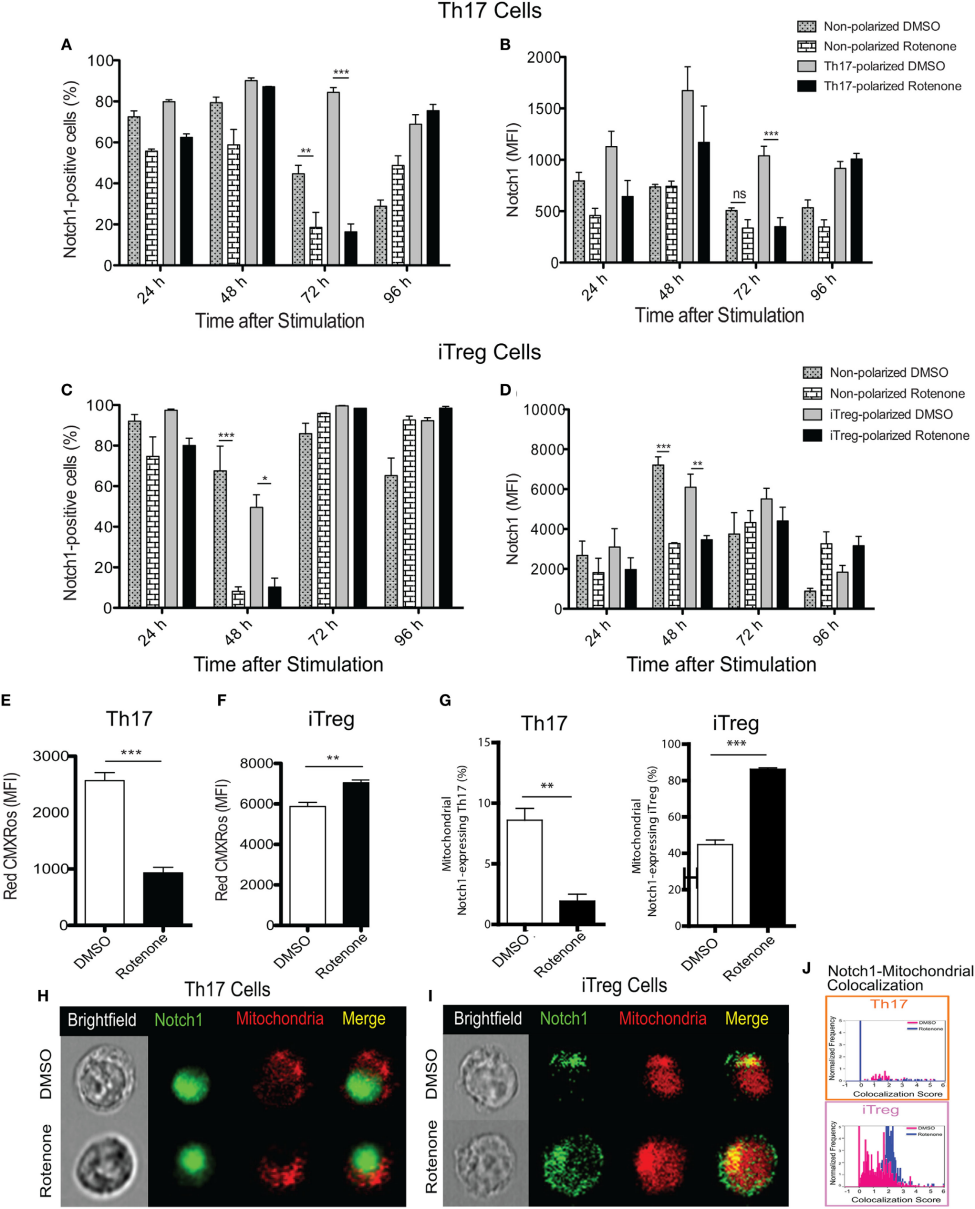
Notch1 expression and cellular localization in T helper (Th) cells is differentially affected by rotenone treatment. Notch1 levels were measured in Th cells, using flow cytometric approaches. Cells were left untreated or treated with 20 µM rotenone for 2 h and then stimulated with plate-bound anti-CD3ε plus anti-CD28 for 24, 48, 72, and 96 h in the presence of specific Th cell polarization conditions. At the indicated timepoints, cells were harvested to determine the **(A)** percent Notch1-positive and **(B)** Notch1 median fluorescence intensity (MFI) in Th17-polarized cells, and the **(C)** percent Notch1-positive, and **(D)** Notch1 MFI in induced regulatory T cell (iTreg)-polarized cells. We visualized mitochondria within the cells using Mitotracker Red CMXRos and imaging flow cytometry. Mitochondrial mass was determined based on the MFI of Red CMXRos for DMSO control and rotenone-treated cells under **(E)** Th17-polarizing conditions 72 h after stimulation and **(F)** iTreg-polarizing conditions 48 h after stimulation. **(G)** We calculated the percent of Th17 (left panel) and iTreg (right panel) cells which showed mitochondrial Notch1 localization. Representative cell images showing Notch1 and mitochondrial colocalization in **(H)** Th17 and **(I)** iTreg cells, differentiated in the presence and absence of rotenone. **(J)** We used the AMNIS Colocalization Wizard to calculate Th17 and iTreg cell frequency histograms that show Notch1 colocalized with mitochondria, together with their corresponding colocalization scores. Data represent the mean ± SEM of three independent experiments. *p < 0.05; **p < 0.01; ***p < 0.001; calculated using two-way ANOVA with post-Bonferroni test applied or an unpaired, two-tailed Student’s *t*-test.

Earlier studies showed Notch1 can regulate IL-17A production in Th17 cells, as well the differentiation of iTreg cells induced *in vitro* (19, 23). Therefore, we asked whether the kinetics of Notch1 expression in each Th cell subset also varied in Th cells in response to rotenone treatment. Using conventional flow cytometry, which quantifies total protein, but not its subcellular localization, we determined the percent of cells that stained positively for Notch1, as well as the total level of Notch1 expressed, in Th1-, Th2-, Th17-, and iTreg-polarized cells. Notch1 expression was significantly diminished in Th17 and iTreg cells, 72 and 48 h after polarization, respectively (Figures 3A–D), displaying response kinetics that overlapped with those of the IL-17A and IL-10 cytokine expression. These data suggest that rotenone may be affecting cytokine secretion in Th17 and iTreg cells through its effects on Notch1. Consistent with this conclusion, we did not find any significant differences in Notch1 expression in Th1 or Th2 cells over time (Figures S2A–D in Supplementary Material).

To further understand the effects of complex I inhibition on Th cell differentiation, we measured the mitochondrial mass in Th cells following rotenone treatment. We also asked whether rotenone treatment influenced the subcellular localization of Notch, by assessing its distribution across mitochondrial, cytosolic, and nuclear compartments. When we measured mitochondrial mass using CMXRos dye, we noted that rotenone treatment significantly reduced the mitochondrial mass in Th1, Th2 (Figures S3A,B in Supplementary Material), and Th17 cells (Figure 3E). By contrast, the mitochondrial mass in iTreg cells was notably increased (Figure 3F). We next quantified the frequency of Th cells that show mitochondrial association of Notch1 using imaging flow cytometry, which can distinguish the subcellular localization of a specific protein of interest. We detected low levels of mitochondrial Notch1 in DMSO-treated Th1, Th2, and Th17 cells (Figures S3C–E in Supplementary Material). Although rotenone treatment did not alter mitochondrial Notch1 colocalization score in Th1 or Th2 cells, the frequency of cells that expressed mitochondrial Notch1 increased with rotenone treatment, and to a much greater extent in Th2 cells (Figures S3C–E in Supplementary Material). We observed almost no mitochondrial-associated Notch1 in Th17 cells, regardless of whether they were differentiated in the presence or absence of rotenone (Figures 3G,H,J). iTregs, however, showed a substantial amount of mitochondrial Notch1 when they were differentiated in the presence of DMSO, and this increased further with rotenone treatment, as determined by the higher colocalization score assigned by the AMNIS Colocalization Wizard algorithm (Figures 3G,I,J). Finally, we applied the AMNIS intensity mask for nucleus, mitochondria, and cytosol to calculate the percent of Notch1 protein residing in these different compartments and determined whether complex I deficiency altered this distribution. We noted slight increases in nuclear Notch1 in rotenone-treated Th1 and iTreg cells, marked decreases in cytosolic Notch1 in Th2 and iTregs, with concomitant increases in mitochondrial Notch1. Most striking was a near complete loss of nuclear Notch1 in Th17 cells, with redistribution to the cytosol (Figure S3E in Supplementary Material). These results suggest that Notch1 localization may be related to cytokine signaling during Th17 and iTreg differentiation and can be altered by ETC-I activity.

### Rotenone Treatment Does Not Affect the Expression of Th Cell Master Transcriptional Regulators

**Figure 4:**
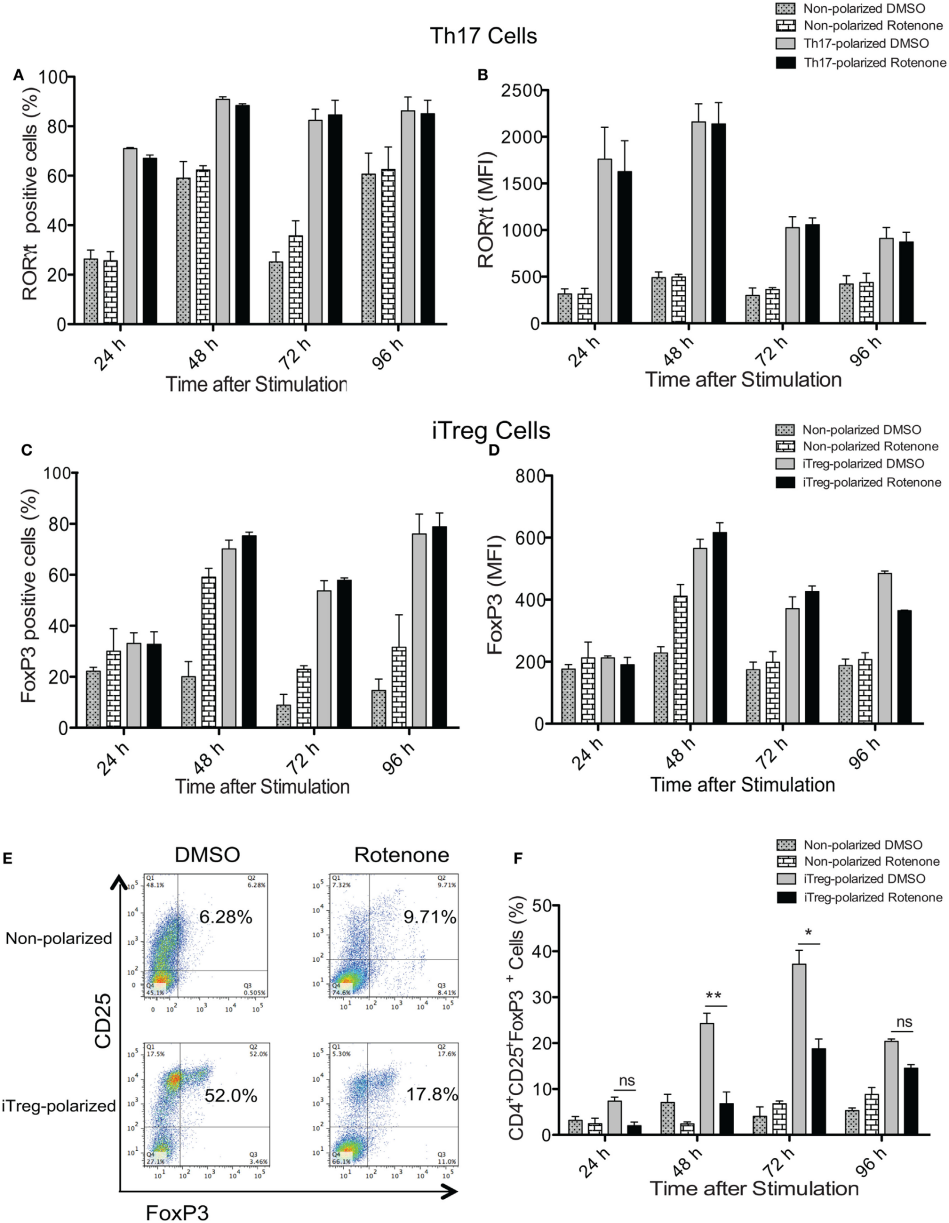
Rotenone treatment does not affect the expression of T helper (Th) cell master transcriptional regulators. CD4 T cells were left untreated or treated with 20 µM rotenone for 2 h and then stimulated with plate-bound anti-CD3ε plus anti-CD28 for 24, 48, 72, and 96 h in the presence of specific Th17- or induced regulatory T cell (iTreg)-specific polarization conditions. At the indicated timepoints, cells were harvested, and we determined the **(A)** percent RORγt-positive and **(B)** median fluorescence intensity (MFI) of RORγt expressed in Th17-polarized cells, and the **(C)** percent Foxp3-positive and **(D)** MFI of Foxp3 expressed in iTreg-polarized cells. **(E)** Representative dot plot and **(F)** collated data showing percentages of CD4^+^CD25^+^Foxp3^+^ iTregs following differentiation in the absence or presence of rotenone. Data represent the mean ± SEM of three independent experiments. *p < 0.05; **p < 0.01; ***p < 0.001; calculated using two-way ANOVA with post-Bonferroni test applied.

Signature Th cytokines are regulated by their master transcription factors. Therefore, we asked whether rotenone also affects the levels of these transcription factors expressed over time. We first assessed levels of RORγt and Foxp3, since changes in Notch1 expression correlated with IL-17A and IL-10 levels during Th17 and iTreg differentiation, respectively. Surprisingly, we did not detect differences in total in RORγt in Th17 cells (Figures 4A,B) or Foxp3 levels in iTreg cells (Figures 4C,D), as measured by flow cytometry, regardless of whether cells were pretreated with rotenone. We did note modest decreases in T-bet levels in Th1-polarized cells (Figures S4A,B in Supplementary Material), but there was no significant effect on GATA3 expression in Th2-polarized cells (Figures S4C,D in Supplementary Material). These results showed that the levels of each transcription factor were not substantially changed after rotenone treatment. However, we found that rotenone treatment decreased the percent of iTregs, characterized as CD4^+^CD25^+^Foxp3^+^ cells, at 48 and 72 h upon iTreg differentiation (Figures 4E,F) suggesting that regulatory T cells rely on their mitochondrial function during the differentiation process.

### Th17 Cells Lose Expression of Nuclear RORγt Following Rotenone Treatment

**Figure 5:**
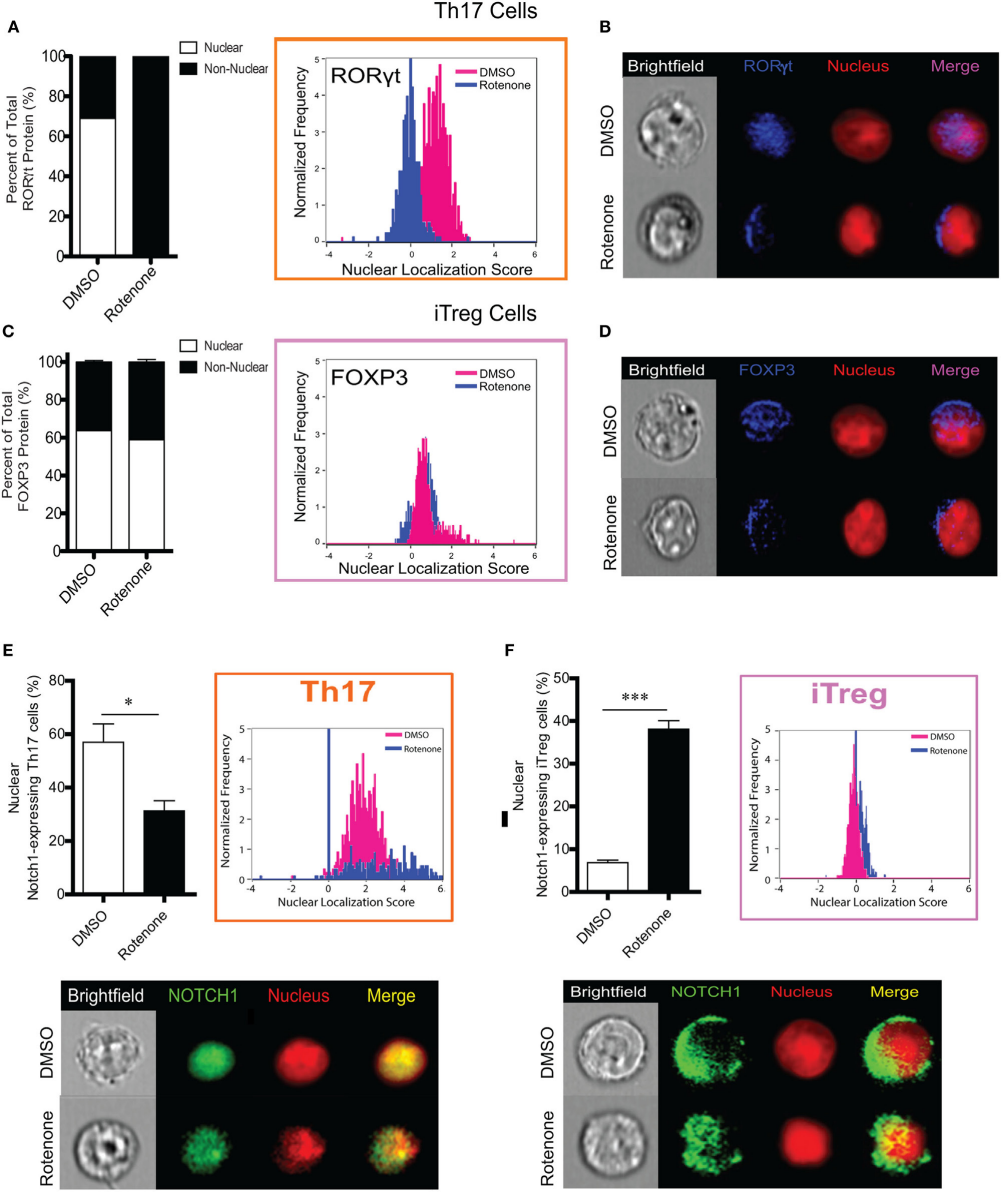
T helper (Th)17 cells lose expression of nuclear RORγt following rotenone treatment. CD4 T cells were left untreated or treated with 20 µM rotenone for 2 h and then stimulated with plate-bound anti-CD3ε plus anti-CD28 for 24, 48, 72, and 96 h in the presence of specific Th17- or induced regulatory T cell (iTreg)-specific polarization conditions. For each master transcriptional regulator, we determined the total, nuclear, and non-nuclear median fluorescence intensity (MFI), using an AMNIS Imaging Flow Cytometer. nuclear localization scores were determined by an algorithm that calculates the probability of the protein of interest resides within the nucleus. The higher the nuclear localization score, the greater the probability of the protein of interest is in the nucleus. Scores above “1” are considered to be positive for nuclear expression. Nuclear and non-nuclear MFI were calculated using AMNIS IDEAS Software and applying the nuclear mask. Percent total nuclear and non-nuclear transcription factor proteins were calculated for cells differentiated in the absence or presence of rotenone as follows: [nuclear MFI]/[Total MFI] × 100, or [non-nuclear MFI]/[Total MFI] × 100, respectively. For cells differentiated without or with rotenone, **(A)** percent distribution of total RORγt protein and RORγt nuclear localization score in Th17-polarized cells. **(B)** Representative images showing nuclear localization of RORγt. **(C)** Percent distribution of total Foxp3 protein and Foxp3 nuclear localization score in iTreg-polarized cells. **(D)** Representative images for nuclear localization of Foxp3 in iTreg cells. For cells differentiated without or with rotenone. **(E)** Percent of nuclear Notch1-expressing Th17 cells and their representative corresponding Nuclear Localization Scores and representative images of Notch1 nuclear localization in Th17 cells after 72 h of differentiation without or with rotenone in Th17 cells 72 h after differentiation, and **(F)** percent of nuclear Notch1-expressing iTreg cells and their representative corresponding Nuclear Localization Scores and representative images of Notch1 nuclear localization in iTreg cells after 48 h of differentiation without or with rotenone. Data represent the mean ± SEM of three independent experiments. *p < 0.05; **p < 0.01; ***p < 0.001; calculated using two-way ANOVA with post-Bonferroni test applied or an unpaired, two-tailed Student’s *t*-test.

One means by which protein activity may be regulated is through selective localization within subcellular compartments. Since we did not detect differences in the total levels of master transcriptional regulators following ETC-I inhibition, we asked whether treating cells with rotenone affected their cellular distribution. We stained differentiated Th cells with antibodies specific for their master transcription factor, together with the nuclear marker, DRAQ5. We then determine the nuclear localization of each of these transcription factors using imaging flow cytometry. We also determined the percent distribution of each transcription factor within and outside of the nucleus by applying the AMNIS nuclear masking wizard. T-bet in Th1 cells and GATA3 in Th2 cells were localized exclusively to the nucleus in DMSO-treated, polarized cells, with only minimal redistribution to the cytosol following rotenone treatment (Figures S5A–D in Supplementary Material). Rotenone treatment had the greatest effect on RORγt localization in Th17 cells, completely abrogating RORγt nuclear localization and concomitantly redirecting it entirely to the cytosol (Figures 5A,B). We expected to see differences in Foxp3 nuclear localization as well, since rotenone treatment had such robust effects on the percentage of CD4^+^CD25^+^Foxp3^+^ cells generated during iTreg differentiation. Interestingly, we found Foxp3 to be similarly distributed between the nucleus and the cytosol, both in DMSO- and in rotenone-treated iTregs (Figures 5C,D). Reports are increasingly linking Notch1 function to Th17–iTreg differentiation axis (33–37); therefore, we also assessed Notch1 localization in both these cell types. We detected nuclear Notch1 in a high percentage of Th17 cells, and the percentage of Th17 cells showing nuclear Notch1 was decreased significantly, following rotenone treatment (Figure 5E). In complementary fashion, fewer than 10% of iTregs expressed nuclear Notch1, but in rotenone-treated iTregs, nuclear Notch1 could be detected in nearly 40% of cells (Figure 5F). Altogether, these results suggest that, although inhibiting ETC-1 with rotenone negatively impacts Th17 and iTreg differentiation *in vitro*, it likely does so through different mechanisms. However, nuclear residence of Notch1 may be necessary for a sustained Th17 differentiation program.

### Rotenone Treatment Inhibits Mitochondrial Localization of PDHK1 and Promotes RORγt and Notch1 Colocalization With Its Phosphorylated Substrate, pPDH-E1α, in Th17-Polarized Cells

**Figure 6:**
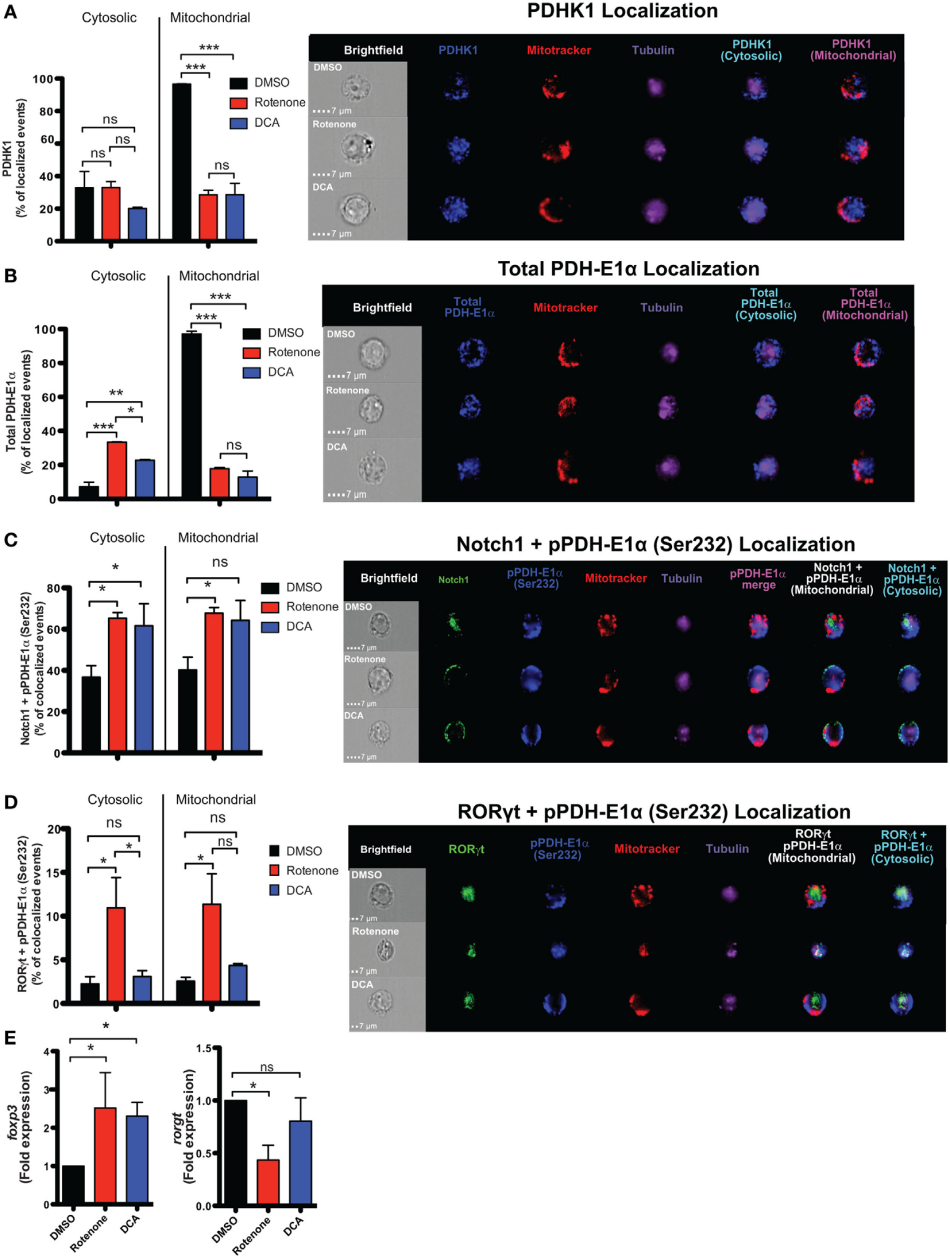
Rotenone treatment inhibits mitochondrial localization of pyruvate dehydrogenase kinase 1 (PDHK1) and promotes RORγt and Notch1 colocalization with its phosphorylated substrate, pPDH-E1α, in Th17-polarized cells. CD4 T cells were treated either with 20 µM rotenone for 2 h or 1 mM dichloroacetate (DCA) (left in the cell suspension throughout) and then stimulated with plate-bound anti-CD3ε plus anti-CD28 for 72 h in the presence of Th17 polarization conditions. After 72 h, samples were split and half of the cells were stained for PDHK1, total PDH-E1α, pPDH-E1α (Ser232), RORγt, and Notch1 to determine their localization in cytosol (Tubulin AF647 staining) and mitochondria (Mitotracker CMXRos). Data were acquired *via* AMNIS ImageStream X Mark Imaging Flow Cytometer. Quantification of percent of cytosolic or mitochondrial **(A)** PDHK1-localizing cells and **(B)** total PDH-E1α-localizing cells along with corresponding representative images are shown. For colocalization, percent of **(C)** Notch1 + pPDH-E1α (Ser232)-colocalizing and **(D)** RORγt + pPDH-E1α (Ser232) Th17-polarized cells for each treatment condition and representative images are shown. Total RNA was collected from the other half of treated cells and qPCR was performed for measuring **(E)** *foxp3* and *rorgt* expressions upon rotenone treatment (DCA treatment was used as a control which triggers *foxp3* expression). Data represent the mean ± SEM of three independent experiments. *p < 0.05; **p < 0.01, ***p < 0.001 calculated using an unpaired, two-tailed Student’s *t*-test.

In the presence of the appropriate cytokine environment, Notch signaling can serve to reinforce Th cell fate decisions, including differentiation into Th17 and iTreg cells (19, 23, 24, 38, 39). Since we observed significantly reduced levels of nuclear Notch1 in Th17 and iTreg-polarized cells, we asked what effects rotenone imparts on Notch1 nuclear localization and its colocalization with master transcription factors. We stained differentiated Th cells with antibodies specific for their signature master transcriptional regulator, together with Notch1, and the nuclear marker DRAQ5, and analyzed colocalization using imaging flow cytometry. Rotenone treatment significantly diminished colocalization of Notch1 with T-bet, GATA3, and RORγt, while Notch1–Foxp3 nuclear colocalization increased in rotenone-treated iTregs (Figures S6A–D in Supplementary Material). Furthermore, data suggest Th17 cells rely heavily on glycolysis, whereas iTregs primarily utilize OXPHOS and FAO, to meet their respective energy needs. Specifically, Gerriets and Kishton identified PDHK1 as a selective regulator of glycolytic and oxidative metabolism by inhibiting PDH (13). PDH inhibition suppressed glucose oxidation and shifted metabolic processes to lactate production and glycolysis (13). The authors found that inhibiting PDHK1 specifically weakened Th17 cells while benefiting iTregs. To further explore how ETC-I integrity affects PDHK1, we generated Th17 CD4 T cells, *in vitro*, in the presence of rotenone or DCA, an inhibitor of PDHK1. DMSO was used as a vehicle control. Cells were stained with a mitochondrial dye, together with antibodies specific for Notch1, RORγt, PDHK1, and its substrate, PDH-E1α, then visualized using imaging flow cytometry. We observed that, in Th17 cells, PDHK1 is localized primarily to the mitochondria (Figure 6A), as is its non-phosphorylated substrate, PDH-E1α (Figure 6B). However, treating cells either with rotenone or DCA diminishes mitochondrial-associated PDHK1 and PDH-E1α, and redistributes PDH-E1α to the cytosol (Figures 6A,B; Figure S7A–C in Supplementary Material). We next asked whether Notch1 or RORγt associated with the phosphorylated form of PDH-E1α, pPDH-E1α (Ser232), in Th17 cells. We observed increases both of cytosolic and mitochondrial Notch1-pPDH-E1α colocalization when Th17 cells were polarized in the presence of rotenone or DCA (Figure 6C; Figure S7D in Supplementary Material). Unexpectedly, when we assessed colocalization of RORγt and pPDH-E1α, we found increased colocalization in Th17 cells only after rotenone treatment (Figure 6D; Figure S7E in Supplementary Material). In addition, colocalization of total PDH-E1α and RORγt did not seem to change in either of the treatments (Figures S7F,G in Supplementary Material), suggesting that, as nuclear RORγt is lost, it may be redistributed and sequestered selectively by phosphorylated form of PDH-E1α [pPDH-E1α (Ser232)] outside of the nucleus in rotenone-treated Th17 cells. These data suggested to us that PDHK1 and, more specifically, its substrate, PDH-E1α, may act as a “rheostat” to regulate Th17–Treg cell fate decisions. Therefore, we examined the effects of rotenone and DCA treatment, both, on the expression of *foxp3* and *rorgt* in Th17 cells. Consistent with this prediction, following treatment with either inhibitor, *foxp3* expression was significantly increased in Th17 cells. However, only in rotenone-treated Th17 cells did we observe decreased *rorgt* expression, suggesting ETC-I integrity is also important for sustained *rorgt* transcription (Figure 6E).

Altogether, we show that using the ETC complex I inhibitor, rotenone, disrupts Th17 and iTreg differentiation, *in vitro*. Furthermore, we demonstrate that, in Th17 cells, rotenone acts to abrogate RORγt nuclear localization and subsequently its colocalization with Notch1 in the nucleus, resulting in diminished IL-17A cytokine production. We conclude from our data, that rotenone decreases the expression of Notch1, the percentage of cells that express nuclear Notch1, and the percentage of Th17 cells that express mitochondrial Notch1, compared with DMSO-treated counterparts, which strongly suggests Notch1 has an integral function in Th17 cell differentiation. We also noted decreased nuclear colocalization of Notch1-RORγt in rotenone-treated Th17 cells, as RORγt is redistributed almost solely to non-nuclear compartments following rotenone treatment, which further suggests that nuclear Notch1 and nuclear RORγt may co-regulate Th17 cell fate. Non-nuclear Notch1 and RORγt, both, associated with pPDH-E1α in rotenone-treated Th17 cells, leading to increased *foxp3* transcription in these cells (Figure S8 in Supplementary Material). These data suggest that the Notch1-RORγt signaling pathway intersects with key regulators of T cell metabolism to influence Th17–iTreg cell fate potential.

## Discussion

Here, we report the effects of inhibiting mitochondrial function on CD4 T cell activation and differentiation. Treating murine splenic CD4 T cells with rotenone, a known ETC-I inhibitor, attenuated T cell activation potential upon anti-CD3ε and anti-CD28 stimulation. This decreased activation was accompanied by reduced secretion of proinflammatory cytokines such as IFNγ and IL-2. We also examined how inhibiting ETC-I function impacts CD4 T cell differentiation into four major Th subsets and showed treatment with rotenone primarily affected Th17 and iTreg differentiation while exhibiting milder effects on Th1 and Th2 cells.

Notch1 localization in Th cell subsets is a critical determinant in their differentiation processes and previous studies have linked Notch signaling to cell survival, based on its mitochondrial versus nuclear association (40, 41). In this study, in iTregs Notch1 localized to the mitochondria, whereas Th17 cells exhibited high levels of nuclear Notch1. Surprisingly, we found Notch1 selectively colocalized with master transcription factors in the nucleus. For instance, RORγt and Notch1 predominantly colocalized in the nucleus and this was abrogated upon rotenone treatment. On the other hand, we did not observe Foxp3 and Notch1 colocalization, although rotenone treatment did reduce the percent of iTregs differentiated in culture. Thus, although rotenone treatment affected both Th17 and iTreg differentiation, through its inhibition of ETC-I function, it is likely that mitochondrial involvement in regulating Th17 and iTreg differentiation is different and distinct in these subsets.

Evidence shows that metabolic reprogramming plays an important role in T cell activation, differentiation, and function (42, 43). As the central hubs of cellular energy production, mitochondria are key regulators of cell metabolism, survival, and signal transduction (44–46). Certain subsets of Th cells rely on OXPHOS to meet their energy demands which, in turn, is directed by a group of proteins found on the outer mitochondrial membrane (47). ATP produced *via* OXPHOS is mediated by the four protein complexes within the ETC, through the creation of a proton gradient. After T cell activation, CD4 T cells undergo a switch from a resting to a proliferating metabolic state, with substantial increases in glucose uptake and in rates of glycolysis reported. This increase in glycolysis is dependent upon CD28-mediated signaling (14). Our results show that the failure to upregulate CD25 and produce IFNγ and IL-2 suggest that rotenone-treated cells do not undergo full TCR-mediated activation. In addition to determining T cell activation, mitochondrial function impacts CD4 T cell differentiation. We measured signature cytokine production in four main Th subsets and found that rotenone treatment reduced Th17 and iTreg cytokine secretion, but not that of Th1 or Th2 cells. One explanation for the differences in sensitivity to ETC-I inhibition could be that Th subsets have intrinsically distinct metabolic characteristics and requirements, especially during the early stages of Th cell commitment.

Data in the literature suggest Th1, Th2, and Th17 cells rely heavily on glycolysis, whereas, iTregs primarily utilize OXPHOS and FAO to meet their respective energy needs. Our findings demonstrate that ETC-I integrity may factor prominently in Th17 and iTreg cell differentiation, and that Notch1 and RORγt do, indeed, converge at the level of cell metabolism in Th17 cells. Gerriets and Kishton identified PDHK1 as a selective regulator of glycolytic and oxidative metabolism by inhibiting PDH (13). PDH inhibition suppresses glucose oxidation and shifts metabolic processes to lactate production and glycolysis (48). Our data are consistent with their findings that inhibiting PDHK1 shifts Th17 cells toward an iTreg phenotype, since we observed increased *foxp3* expression in Th17 cells treated either with rotenone or the PDHK1 inhibitor, DCA. We further add the critical mechanistic observation that PDHK1 activity is linked to ETC-I function during Th17 differentiation, by altering the cellular distribution of pPDH-E1α, as well as its association with Notch1 and RORγt.

There is a growing body of literature that identifies a Notch1-regulated “switch” between Th17 and iTreg cells (33–37). Our findings are consistent with a function for nuclear Notch1, together with nuclear RORγt, in promoting Th17 differentiation and suggest that defects in ETC-I function alter Notch1, as well as RORγt, nuclear localization leading to reduced cytokine production. How mitochondrial function and protein localization are linked is not entirely clear. In macrophages, Notch1 has been shown to be recruited to the promoters of nitric oxide synthase 2 (*Nos2*) and pyruvate dehydrogenase phosphatase 1 (*Pdp1*) genes to mediate cell activation and mitochondrial glucose oxidation, respectively. PDP1 functions in opposition to PDHK1, suggesting that PDH activation may promote iTreg differentiation. Moreover, Notch1 signaling coordinates both components of OXPHOS: TCA cycle and ETC (25). For instance, one report demonstrated that, in response to Notch1 activation, the mitochondrial proteome is altered, thus impacting both the TCA and ETC in mitochondria (49). Hyperactivation of Notch1 signaling accelerates glycolysis by activating the phosphatidylinositol 3-kinase/AKT pathway, whereas hypoactivation of Notch1 signaling attenuated mitochondrial activity and induced glycolysis. However, only when Notch1 had been hyperactivated could cells switch back to OXPHOS (26). At this stage, then, Notch1 localization to mitochondria may be essential to modulating specific metabolic pathways and, thus, intersect with distinct Th cell differentiation programs.

Our data show that Notch1 can colocalize with RORγt in the nucleus and nuclear localization of RORγt, but not of Foxp3, is abrogated upon rotenone treatment. Although we did not find any difference in Notch1-Foxp3 colocalization in the nucleus, mitochondrial Notch1 localization was significantly increased in rotenone-treated iTregs. These data may support the notion that Notch1 in mitochondria can negatively impact iTreg differentiation and act independently of Foxp3 regulation. We detected negligible amounts of mitochondrial Notch1 or RORγt in Th17 cells differentiated in DMSO or in rotenone, suggesting these proteins are not resident in the mitochondria in Th17 cells. However, a pool of mitochondrial-associated STAT3 has been shown to associate within and enhance the functions of the ETC-I (50). A recent report has identified an IL-6-STAT3-dependent pathway that maintains mitochondrial membrane potential, keeps intracellular Ca^++^ stores high, and is necessary in the late stages of Th17 differentiation (51). It is interesting to speculate that the decreased mitochondrial mass, observed in rotenone-treated Th17 cells, may also impact this pool of STAT3 to further negatively regulate Th17 polarization, although this awaits further exploration.

Cancer cells require intact mitochondrial activity during tumor progression (52), identifying mitochondrial complex I as an attractive therapeutic target. Altogether, we have demonstrated that using rotenone to inhibit electron complex I function decreases CD4 T cell activation and impacts Th17 and iTreg differentiation *in vitro*, through its effects on the cellular distribution of Notch1 and RORγt, as well as on key components of cellular metabolism, PDHK1, and its substrate, PDH-E1α. Our results implicate mitochondrial function as a critical contributing factor in the proper differentiation of Th17 and iTreg cells and may pave the way for further examination of mitochondrial deficiency as a regulator of immune dysfunction, especially in the context of tumor therapy to target mitochondrial metabolism.

## Author Contributions

EO, HS, VM, GT, and AL acquired and analyzed data. GNT, NY, and LM provided scientific input. LM conceived of the experimental plan and wrote the manuscript together with EO.

## Conflict of Interest Statement

The authors declare that the research was conducted in the absence of any commercial or financial relationships that could be construed as a potential conflict of interest.
