# Supplementary material for: Rotenone Treatment Reveals a Role for Electron Transport Complex I in the Subcellular Localization of Key Transcriptional Regulators During T Helper Cell Differentiation
Source: Front Immunol. 2018 Jun 7;9:1284. doi: 10.3389/fimmu.2018.01284 (PMC5999735; doi:10.3389/fimmu.2018.01284)
Supplement: Supplementary file 1 [file data_sheet_1.PDF]

Supplementary Material

Figure S1. Rotenone treatment does not affect cellular viability during Th cell differentiation. We used 7-Aminoactinomycin (7-AAD) staining to determine cellular viability of CD4 T cells left untreated or treated with 20 μM rotenone for 2 hours and then stimulated with plate-bound anti-CD3ε plus anti-CD28 for 24, 48, 72, and 96 hours in the presence of specific Th cell polarization conditions. Viability is shown for (**A**) Th1 cells, (**B**) Th2 cells, (**C**) Th17 cells, and (**D**) iTreg cells. Data represent the mean + SEM of three independent replicates.

Figure S2. Rotenone treatment does not affect Notch1 expression in Th1 and Th2 cells. Notch1 levels were measured in Th cells, using flow cytometric approaches. CD4 T cells were left untreated or treated with 20 μM rotenone for 2 hours and then stimulated with plate-bound anti-CD3ε plus anti-CD28 for 24, 48, 72, and 96 hours in the presence of specific Th cell polarization conditions. At the indicated timepoints, cells were harvested and we determined the (**A**) percent Notch1-positive and (**B**) Notch1 MFI in Th1-polarized cells, and the (**C**) percent Notch1-positive, and (**D**) Notch1 MFI in Th2-polarized cells.

Figure S3. Rotenone reduces mitochondrial mass in Th1 and Th2 cells and alter mitochondrial Notch1 localization only in Th2 and iTreg cells. CD4 T cells were left untreated or treated with 20 μM rotenone for 2 hours and then stimulated with plate-bound anti-CD3ε plus anti-CD28 for 24, 48, 72, and 96 hours in the presence of specific Th cell polarization conditions. We stained mitochondria for each cell type by using Mitotracker Red CMXRos and measured the intensity by imaging flow cytometry. Mitochondrial mass was determined by the median fluorescent intensity of Red CMXRos for DMSO control and rotenone-treated cells under (**A**) Th1-polarized conditions at 72 hours after stimulation, (**B**) Th2-polarized conditions at 48 hours after stimulation, (**C**) Th1 and Th2 cell frequency graphs and representative histograms that have colocalization of Notch1 at the mitochondria with their corresponding colocalization scores determined by AMNIS software. (**D**) Representative cell images of Notch1-mitochondrial colocalization in Th1 and Th2 cells. (**E**) Notch1 distribution in subcellular compartments in four different Th cell subsets calculated by AMNIS software. Data represent the mean + SEM of three independent experiments. *$p<0.05$; **$p<0.01$, ***$p<0.001$ calculated using an unpaired, two-tailed Student's *t*-test.

Figure S4. Expression of master transcription factors for Th1 and Th2 cell differentiated in the absence or presence of rotenone. CD4 T cells were left untreated or treated with 20 μM rotenone for 2 hours and then stimulated with plate-bound anti-CD3ε plus anti-CD28 for 24, 48, 72, and 96 hours in the presence of specific Th cell polarization conditions. (**A**) Percent T-bet-positive and (**B**) MFI of T-bet expression in Th1-polarized cells. and (**C**) percent GATA3-positive and (**D**) MFI of GATA3 expression in Th2-polarized cells was measured by flow cytometry. Data are the mean + SEM of three independent replicates.

Figure S5. Protein distribution profiles of Th-specific master transcription factors after rotenone treatment. CD4 T cells were left untreated or treated with 20 μM rotenone for 2 hours and then stimulated with plate-bound anti-CD3ε plus anti-CD28 for 24, 48, 72, and 96 hours in the presence of specific Th1 or Th2 cell polarization conditions. We determined the median fluorescent intensity of specific master transcription factors both within (Nuclear MFI) and outside of the nucleus (Non-Nuclear MFI) by Imaging Flow Cytometry using AMNIS IDEAS software. We calculated the percent of total T-bet protein within and outside of the nucleus using the following equations, respectively: [Nuclear MFI] / [Total MFI] x 100 and [Non-Nuclear MFI] / [Total MFI] x 100. **(A)** Percent of T-bet protein with representative histograms and images for T-bet nuclear localization in Th1-polarized cells and for (**B**) GATA-3 nuclear localization in Th2-polarized cells. **(C)** Th1 and Th2 cell frequency graphs and representative histograms that have localization of Notch1 in the nucleus. (**D**) Representative cell images of Notch1 nuclear colocalization in Th1 and Th2 cells. Data represent the mean + SEM of three independent experiments. *$p<0.05$; **$p<0.01$, ***$p<0.001$ calculated using an unpaired, two-tailed Student's *t*-test.

Figure S6. Rotenone treatment alters colocalization of Notch1 with Th master transcription factors. CD4 T cells were left untreated or treated with 20 μM rotenone for 2 hours and then stimulated with plate-bound anti-CD3ε plus anti-CD28 for 24, 48, 72, and 96 hours in the presence of specific Th1, Th2, Th17, or iTreg cell polarization conditions. We determined the median fluorescent intensity of Notch1 or of specific master transcription factors both within (Nuclear MFI) and outside of the nucleus (Non-Nuclear MFI) by Imaging Flow Cytometry using AMNIS IDEAS software. Cell frequency data, cumulative histograms, and colocalization scores with representative images for (**A**) Notch1-T-bet colocalization in Th1 cells after 72 hours of differentiation, for **(B)** Notch1-GATA3 colocalization in Th2 cells after 48 hours of differentiation, for **(C)** Notch1-RORγt colocalization in Th17 cells after 72 hours of differentiation, and for **(D)** Notch-FOXP3 colocalization in iTreg cells after 48 hours of differentiation. Data represent the mean + SEM of three independent experiments. *$p<0.05$; **$p<0.01$, ***$p<0.001$ calculated using an unpaired, two-tailed Student's *t*-test.

Figure S7. Effects of rotenone in PDHK1-RORγt-Notch1 axis in Th17-polarized cells. CD4 T cells were treated either with 20 μM rotenone for 2 hours or 1 mM DCA (left in the cell suspension throughout) and then stimulated with plate-bound anti-CD3ε plus anti-CD28 for 72 hours in the presence of Th17 polarization conditions. Cells were stained for PDHK1, total PDH-E1α, pPDH-E1α (Ser232), RORγt, and Notch1 to determine their localization in the cytosol (Tubulin AF647 staining) and mitochondria (Mitotracker CMXRos). Data were acquired using an AMNIS ImageStream X Mark Imaging Flow Cytometer. Frequency histograms, along with their localization scores, are shown for cytosolic and mitochondrial **(A)** PDHK1, **(B)** Total PDH-E1α, and **(C)** pPDH-E1α (Ser232) localization. For colocalization, frequency histograms, along with their localization scores, are shown for **(D)** Notch1 + pPDH-E1α (Ser232), **(E)** RORγt + pPDH-E1α (Ser232), and **(F)** RORγt + Total PDH-E1α colocalization. **(G)** Percent of RORγt + Total-PDH-E1α colocalizing Th17-polarized cells for each treatment condition and representative images. Data represent the mean + SEM of three independent experiments.

Figure S8. Graphical representation of PDHK1-RORγt-Notch1 localization in Th17-polarized cells under different treatment conditions. **(A)** Notch1 and RORγt colocalize in the nucleus during Th17 differentiation. The metabolic enzyme, PDHK1, and its non-phosphorylated substrate, PDH-E1α, are found primarily in the mitochondria in Th17 cells. **(B)** Rotenone treated Th17 cells show, decreased nuclear Notch1 and RORγt that is redistributed to non-nuclear compartments, where it can be found in association with pPDH-E1α. **(C)** When Th17 cells are treated with the PDHK1 inhibitor, DCA, Notch1 shows increased association with pPDH-E1α in the cytosol and at the mitochondria, however, this same effect is not seen with RORγt.

Figure S1

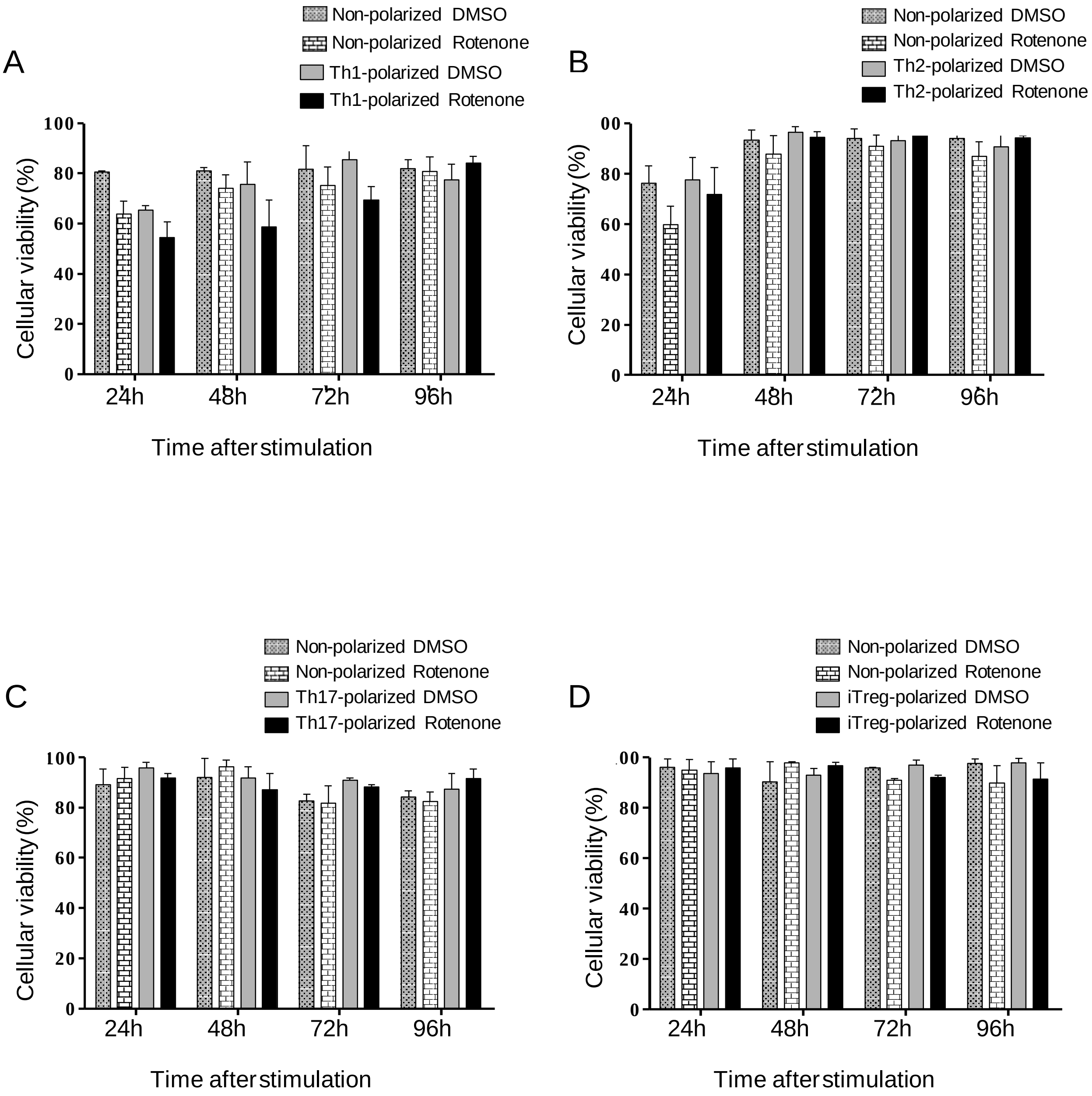
A
Non-polarized DMSO
Non-polarized Rotenone
Th1-polarized DMSO
Th1-polarized Rotenone
Cellular viability(%)
0
20
40
60
80
100
24h
48h
72h
96h
Time after stimulation
B
Non-polarized DMSO
Non-polarized Rotenone
Th2-polarized DMSO
Th2-polarized Rotenone
Cellular viability(%)
Time after stimulation
C
Non-polarized DMSO
Non-polarized Rotenone
Th17-polarized DMSO
Th17-polarized Rotenone
Cellular viability(%)
Time after stimulation
D
Non-polarized DMSO
Non-polarized Rotenone
iTreg-polarized DMSO
iTreg-polarized Rotenone
Cellular viability(%)
Time after stimulation

Figure S2

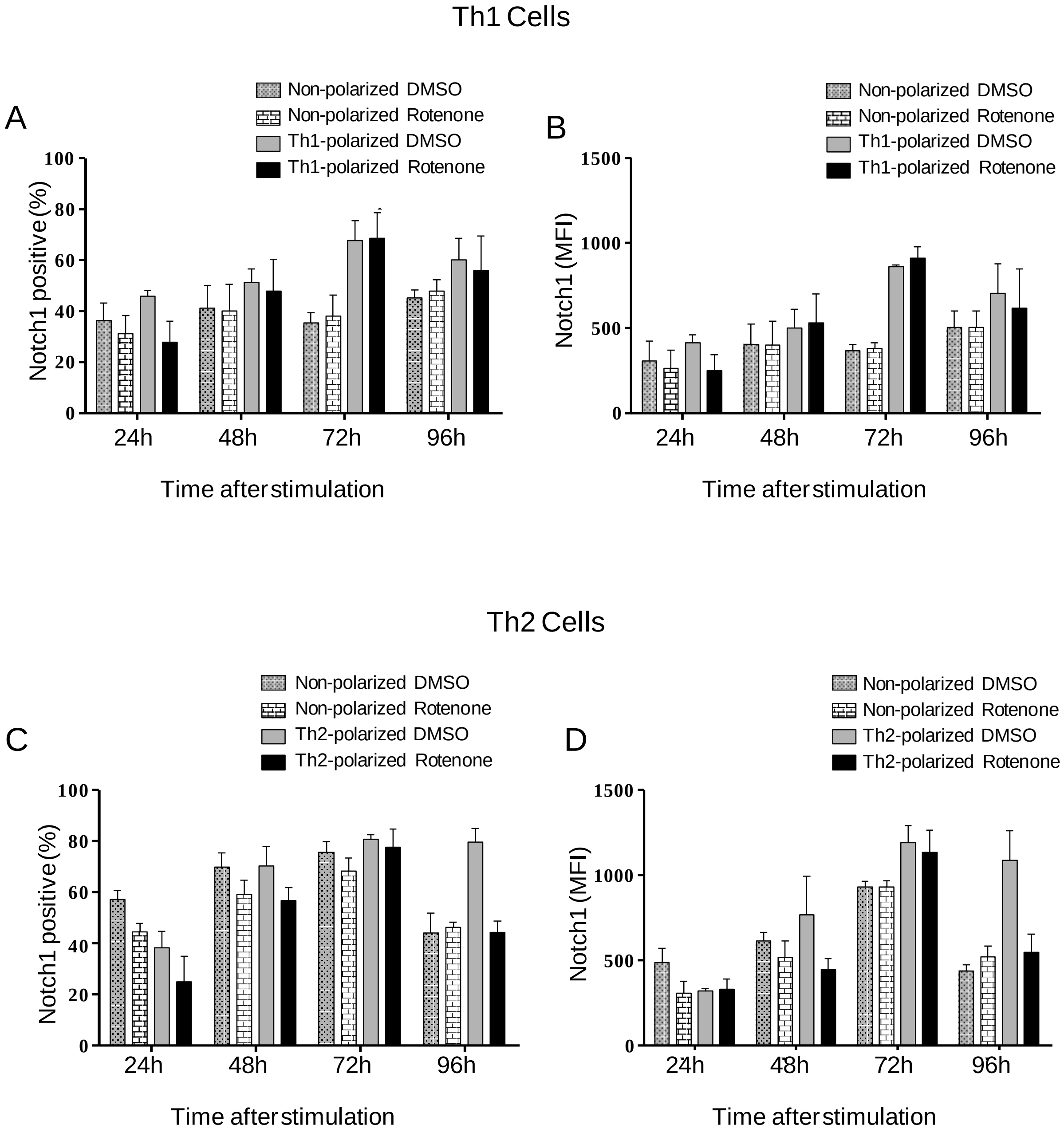
Th1 Cells
A
Non-polarized DMSO
Non-polarized Rotenone
Th1-polarized DMSO
Th1-polarized Rotenone
Notch1 positive (%)
100
80
60
40
20
0
24h
48h
72h
96h
Time after stimulation
B
Non-polarized DMSO
Non-polarized Rotenone
Th1-polarized DMSO
Th1-polarized Rotenone
Notch1 (MFI)
1500
1000
500
0
24h
48h
72h
96h
Time after stimulation
Th2 Cells
C
Non-polarized DMSO
Non-polarized Rotenone
Th2-polarized DMSO
Th2-polarized Rotenone
Notch1 positive (%)
100
80
60
40
20
0
24h
48h
72h
96h
Time after stimulation
D
Non-polarized DMSO
Non-polarized Rotenone
Th2-polarized DMSO
Th2-polarized Rotenone
Notch1 (MFI)
1500
1000
500
0
24h
48h
72h
96h
Time after stimulation

Figure S3

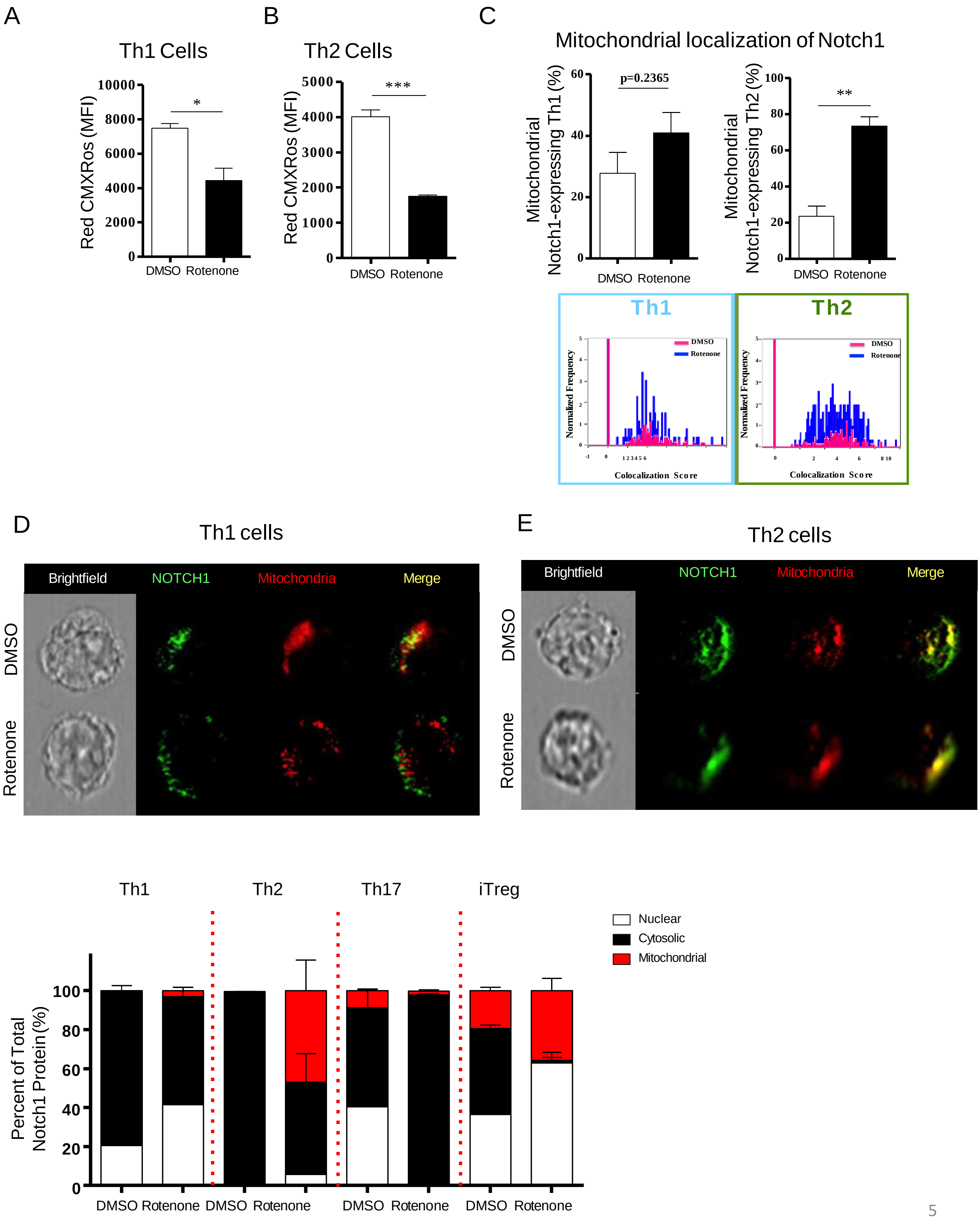
A
Th1 Cells
Red CMXRos (MFI)
10000
8000
6000
4000
2000
0
*
DMSO Rotenone
B
Th2 Cells
Red CMXRos (MFI)
5000
4000
3000
2000
1000
0
***
DMSO Rotenone
C
Mitochondrial localization of Notch1
Mitochondrial Notch1-expressing Th1 (%)
60
40
20
0
p=0.2365
DMSO Rotenone
Mitochondrial Notch1-expressing Th2 (%)
100
80
60
40
20
0
**
DMSO Rotenone
Th1
Normalized Frequency
DMSO
Rotenone
Colocalization Score
Th2
Normalized Frequency
DMSO
Rotenone
Colocalization Score
D
Th1 cells
Brightfield
NOTCH1
Mitochondria
Merge
DMSO
Rotenone
E
Th2 cells
Brightfield
NOTCH1
Mitochondria
Merge
DMSO
Rotenone
Th1
Th2
Th17
iTreg
Nuclear
Cytosolic
Mitochondrial
Percent of Total Notch1 Protein (%)
100
80
60
40
20
0
DMSO Rotenone DMSO Rotenone DMSO Rotenone DMSO Rotenone

Figure S4

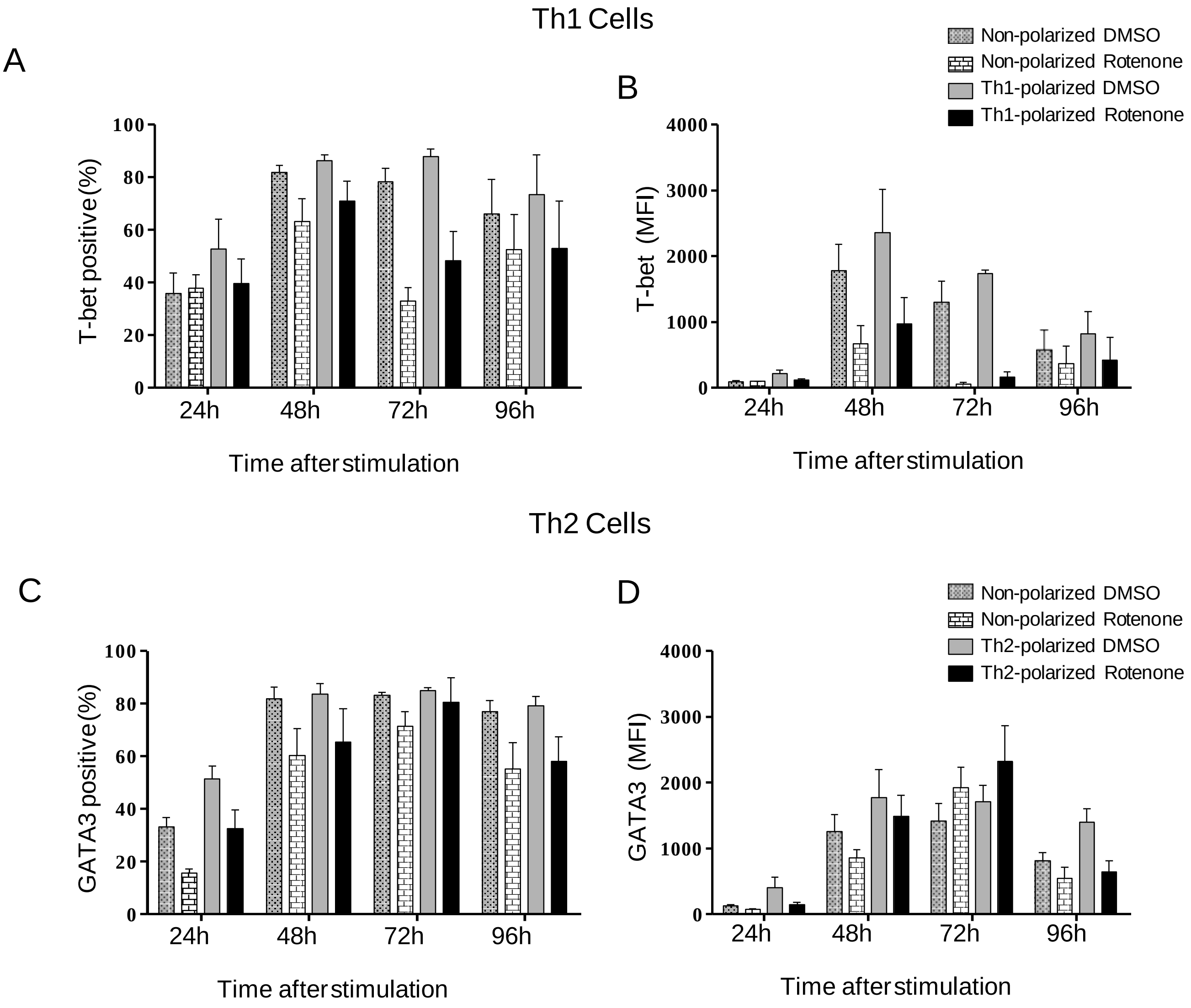
Th1 Cells
A
B
Non-polarized DMSO
Non-polarized Rotenone
Th1-polarized DMSO
Th1-polarized Rotenone
T-bet positive (%)
0
20
40
60
80
100
24h
48h
72h
96h
Time after stimulation
T-bet (MFI)
0
1000
2000
3000
4000
24h
48h
72h
96h
Time after stimulation
Th2 Cells
C
D
Non-polarized DMSO
Non-polarized Rotenone
Th2-polarized DMSO
Th2-polarized Rotenone
GATA3 positive (%)
0
20
40
60
80
100
24h
48h
72h
96h
Time after stimulation
GATA3 (MFI)
0
1000
2000
3000
4000
24h
48h
72h
96h
Time after stimulation

Figure S5

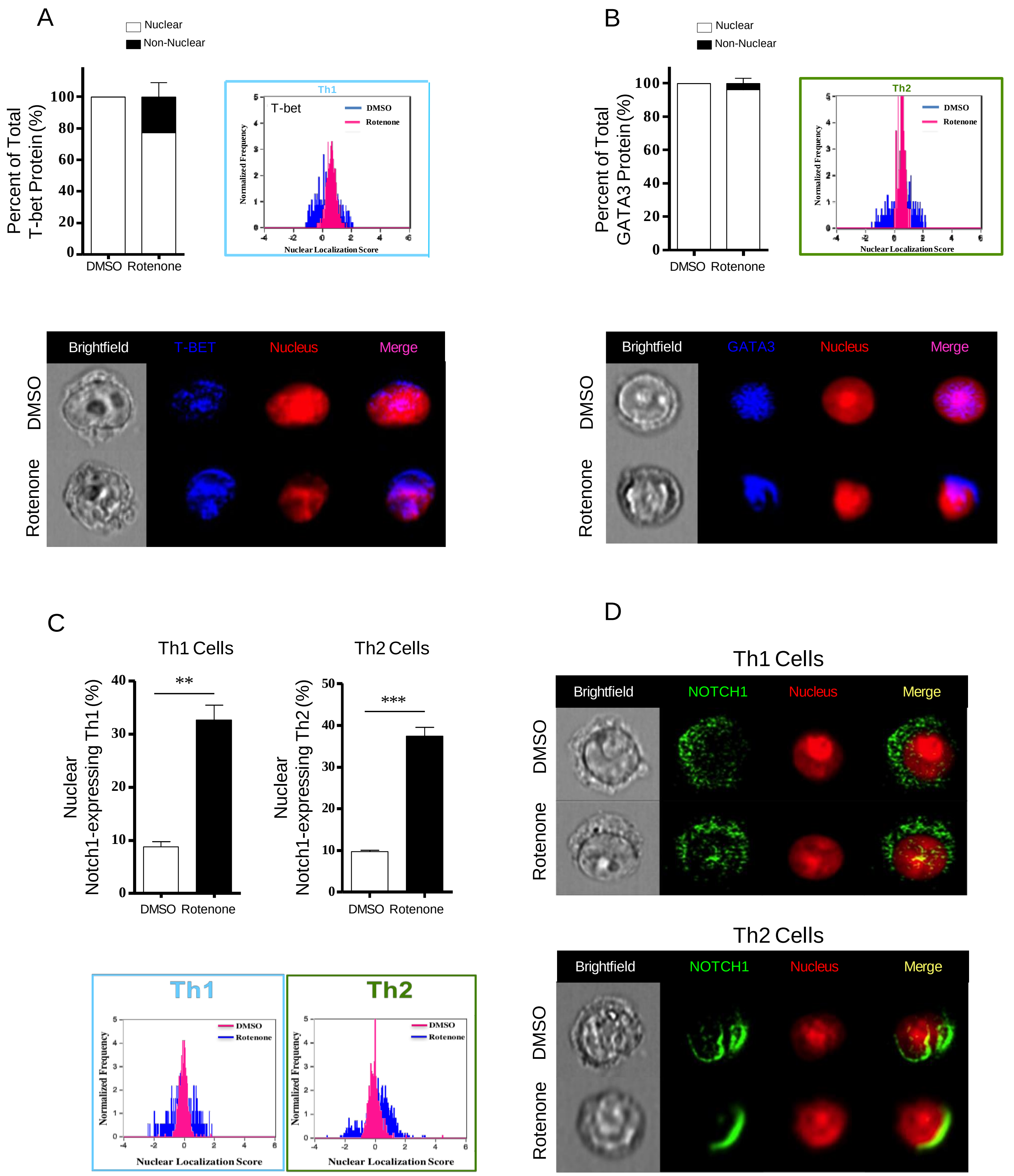

A
Nuclear
Non-Nuclear
Percent of Total T-bet Protein (%)
DMSO
Rotenone
Th1
T-bet
Normalized Frequency
Nuclear Localization Score
Brightfield
T-BET
Nucleus
Merge
B
GATA3 Protein (%)
Th2
GATA3
C
Th1 Cells
Th2 Cells
Nuclear Notch1-expressing Th1 (%)
Nuclear Notch1-expressing Th2 (%)
**
***
D
NOTCH1

Figure S6

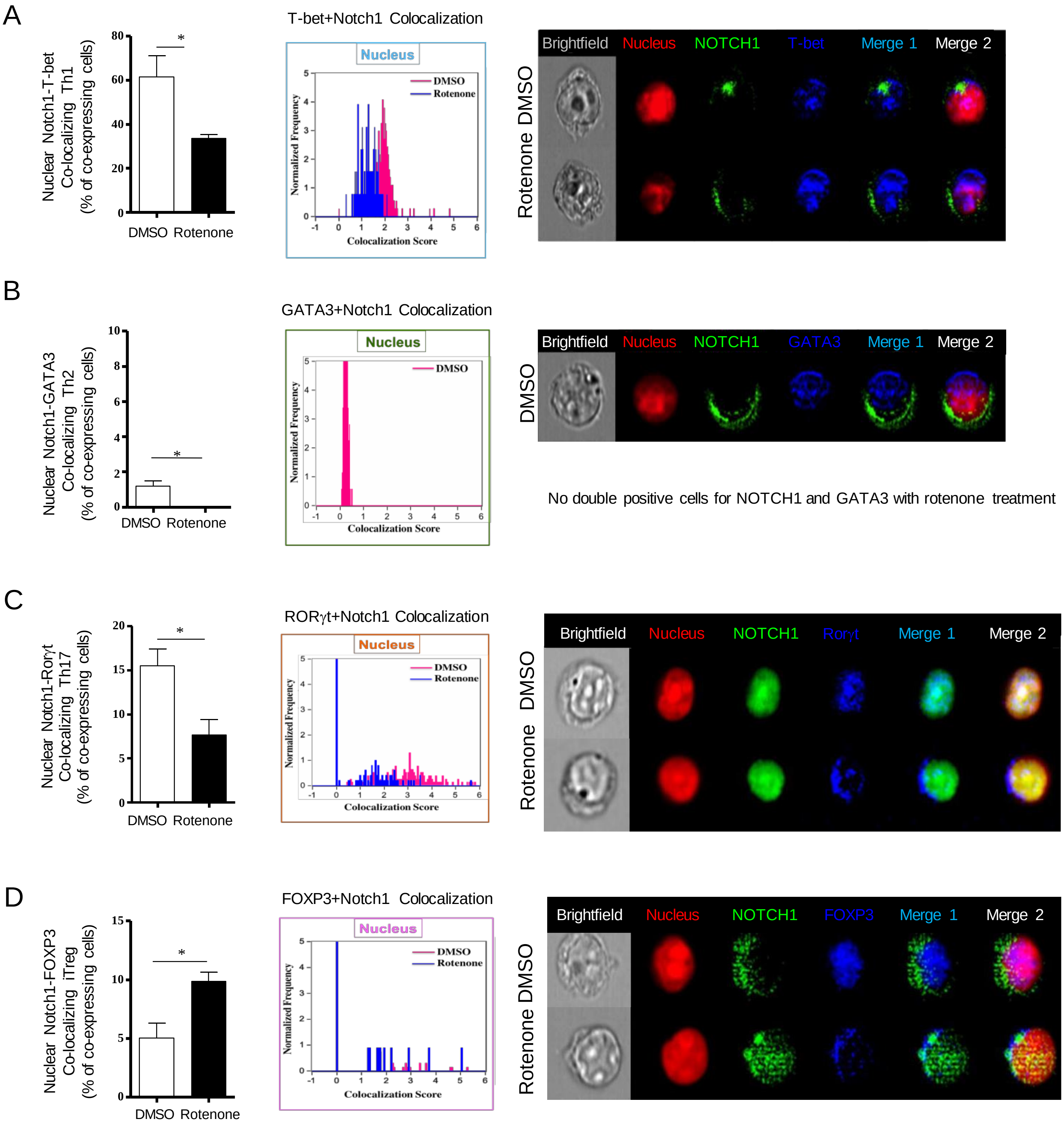
A
Nuclear Notch1-T-bet Co-localizing Th1 (% of co-expressing cells)
DMSO Rotenone
T-bet+Notch1 Colocalization
Nucleus
Normalized Frequency
Colocalization Score
DMSO
Rotenone
Brightfield Nucleus NOTCH1 T-bet Merge 1 Merge 2
Rotenone DMSO
B
Nuclear Notch1-GATA3 Co-localizing Th2 (% of co-expressing cells)
DMSO Rotenone
GATA3+Notch1 Colocalization
Nucleus
DMSO
Brightfield Nucleus NOTCH1 GATA3 Merge 1 Merge 2
No double positive cells for NOTCH1 and GATA3 with rotenone treatment
C
Nuclear Notch1-Rorγt Co-localizing Th17 (% of co-expressing cells)
DMSO Rotenone
RORγt+Notch1 Colocalization
Nucleus
Brightfield Nucleus NOTCH1 Rorγt Merge 1 Merge 2
Rotenone DMSO
D
Nuclear Notch1-FOXP3 Co-localizing iTreg (% of co-expressing cells)
DMSO Rotenone
FOXP3+Notch1 Colocalization
Nucleus
Brightfield Nucleus NOTCH1 FOXP3 Merge 1 Merge 2
Rotenone DMSO

Figure S7

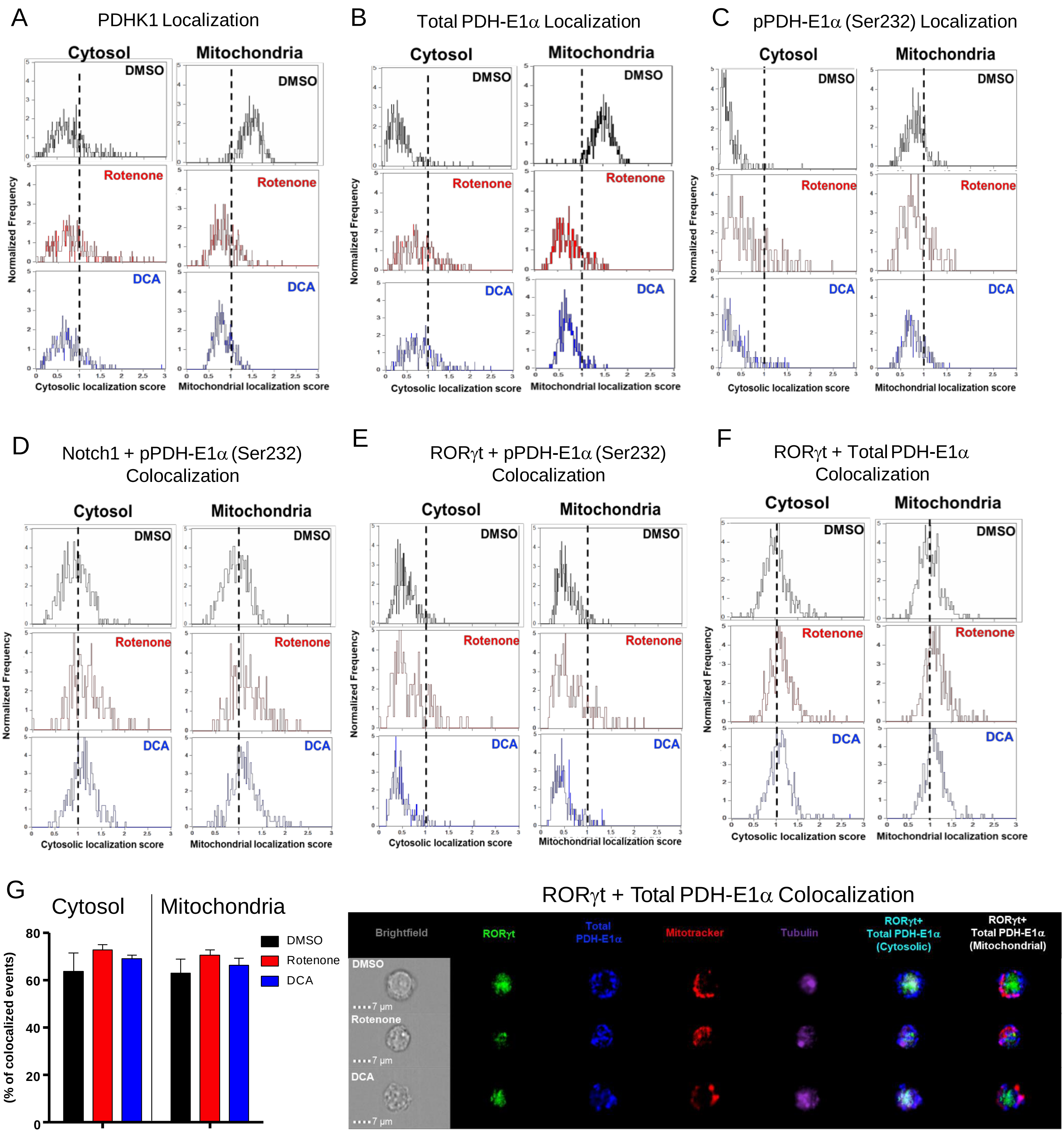

A
PDHK1 Localization
B
Total PDH-E1α Localization
C
pPDH-E1α (Ser232) Localization
Cytosol
Mitochondria
DMSO
Rotenone
DCA
Normalized Frequency
Cytosolic localization score
Mitochondrial localization score
D
Notch1 + pPDH-E1α (Ser232) Colocalization
E
RORγt + pPDH-E1α (Ser232) Colocalization
F
RORγt + Total PDH-E1α Colocalization
G
RORγt + Total PDH-E1α (% of colocalized events)
RORγt + Total PDH-E1α Colocalization
Brightfield
RORγt
Total PDH-E1α
Mitotracker
Tubulin
RORγt+ Total PDH-E1α (Cytosolic)
RORγt+ Total PDH-E1α (Mitochondrial)
7 μm

Figure S8

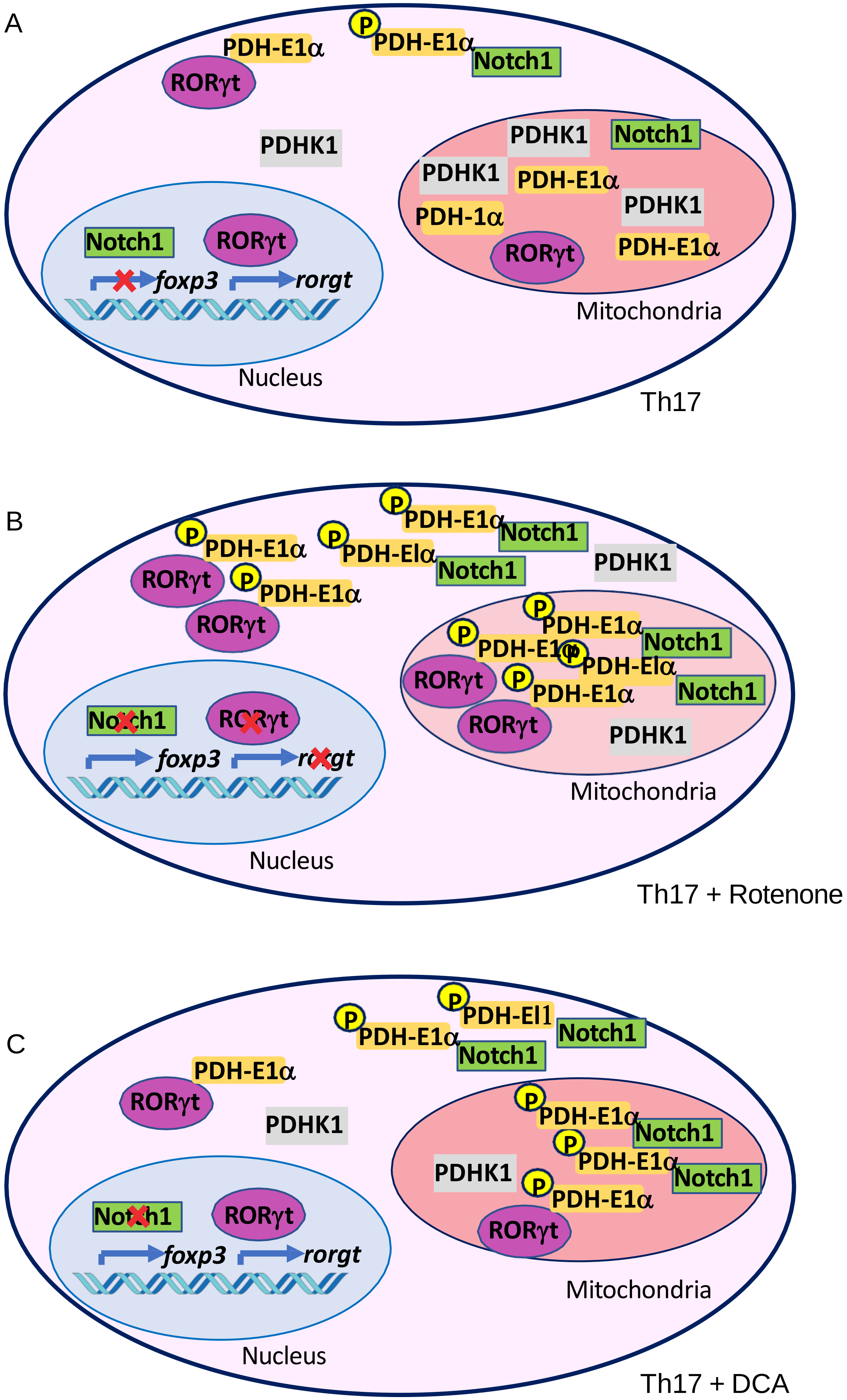
A
P
PDH-E1α
Notch1
RORγt
PDHK1
Notch1
PDH-1α
foxp3
rorgt
Mitochondria
Nucleus
Th17
B
P
PDH-Elα
PDHK1
Mitochondria
Nucleus
Th17 + Rotenone
C
PDH-El1
Mitochondria
Nucleus
Th17 + DCA
